# Risk of Early Childhood Dental Caries Associated With Prolonged Breastfeeding: A Systematic Review and Meta‐Analysis

**DOI:** 10.1111/ipd.13313

**Published:** 2025-04-20

**Authors:** Karina Lustosa, Larissa Rosa Santana Rodrigues, Reuber Mendes Rocha, Tiago Paiva Prudente, Eleazar Mezaiko, Fernanda Paula Yamamoto Silva, Brunno Santos Freitas Silva

**Affiliations:** ^1^ Department of Oral Medicine University of Anápolis Anápolis GO Brazil; ^2^ Department of Stomatologic Sciences, School of Dentistry Federal University of Goiás Goiânia GO Brazil; ^3^ Universidade Federal de Goiás Goiânia GO Brazil; ^4^ University of Anápolis Anápolis GO Brazil

**Keywords:** dental education, prevention, syndromes head and neck/cleft lip and palate

## Abstract

**Background:**

Breastfeeding provides essential nutrients and benefits for newborns. However, its prolonged duration has raised concerns about potential risks for early childhood caries (ECC).

**Aim:**

To determine if prolonged breastfeeding increases the risk of dental caries in children under 71 months.

**Design:**

Eligibility criteria included observational studies comparing ECC risk in children breastfed for over 12 months, with no language restrictions. Databases searched included PubMed, Scopus, and others, up to May 17, 2024. Risk of bias was assessed using the JBI Critical Appraisal Checklist. Meta‐analyses were performed using a random‐effects model.

**Results:**

Twenty‐five studies involving 19 681 participants were included. Studies showed an increased risk of ECC in children breastfed for more than 24 months (RR = 2.44; 95% CI, 1.97 to 3.02). For the 12–24 months period, no significant risk increase was found. Meta‐analyses also indicated higher ECC prevalence with breastfeeding beyond 12 months (OR = 1.86; 95% CI, 1.48 to 2.35).

**Conclusion:**

Prolonged breastfeeding beyond 24 months is associated with an increased risk of ECC. This review highlights the need for future studies to address current research limitations and better understand the relationship between prolonged breastfeeding and ECC.

**Trial Registration:**

International Prospective Register of Systematic Reviews (PROSPERO): CRD42024509212

**Summary**

- Why this paper is important to pediatric dentists
  ○ This SR highlights a significant association between prolonged breastfeeding (beyond 24 months) and an increased risk of ECC, indicating the need for careful monitoring of dental health in children who are breastfed for extended periods.
  ○ Provides clarity on the specific risk factors associated with prolonged breastfeeding (> 12 months) and their implications for ECC, aiding in targeted preventive strategies and patient education.
  ○ The findings suggest that while breastfeeding up to 12 months may be beneficial, extended breastfeeding beyond 24 months could increase the risk of ECC, prompting pediatric dentists to consider this when advising parents in the context of WHO and UNICEF's breastfeeding recommendations.

## 1 Introduction

Breastfeeding is recognized as a rich source of high‐quality nutrients for newborns, offering a wide range of benefits for development, growth, and immunity [1]. The World Health Organization (WHO) and the United Nations Children's Fund (UNICEF) recommend exclusive breastfeeding for the first 6 months of a child's life, followed by the introduction of appropriate complementary foods, with continued breastfeeding up to 24 months or beyond [2, 3]. Despite the well‐documented benefits of breastfeeding, there are concerns regarding its potential risk for dental caries. A recent birth cohort study conducted in Brazil found an association between prolonged breastfeeding and early childhood caries (ECC) at 48 months. The results suggested that breastfeeding beyond 24 months of age might have negative implications for dental health [1].

ECC is defined as the presence of one or more decayed, missing, or filled tooth surfaces in primary teeth in children younger than 71 months (approximately 6 years) [4]. ECC remains a significant health issue worldwide and is a major contributor to health inequities [5]. Several risk factors contribute to ECC, including the quality of tooth brushing, parental supervision, brushing frequency, fluoride exposure, dietary habits, and breastfeeding patterns [6].

Regarding breastfeeding and the risk of ECC, several systematic reviews (SRs) have addressed this topic over the years [7, 8, 9, 10, 11, 12], presenting conflicting evidence in a context of methodological inconsistency. Valaitis et al. [7], in 2000, did not confirm a pooled relationship between ECC and breastfeeding. They observed inconsistent definitions of ECC in the included studies, lack of prospective design, and lack of control for relevant variables such as dental health practices. In 2015, Avila et al. [8], based on pooled evidence, concluded that breastfeeding can protect against ECC, with a lower risk of ECC compared to bottle‐fed children. Tham et al. [9] systematically assessed the associations between breastfeeding and dental caries within specific time frames of childhood. They found that breastfeeding up to 12 months of age reduced the risk of caries, but there was an increased risk in children breastfed for more than 12 months. Cui et al. [10], in 2017, performed an analysis of case–control and cohort studies regarding the risk of ECC. They suggested that breastfeeding might protect children from ECC but could become a risk factor for ECC when the duration of breastfeeding extends beyond 12 months. Similarly, Moynihan et al. [11] conducted a SR addressing various questions about the risk of ECC in children breastfed at different ages. Regarding whether breastfeeding beyond 2 years increases the risk of ECC compared to breastfeeding for less than 2 years, they analyzed two cohort studies, one case–control study, and five cross‐sectional studies. Their findings indicated that breastfeeding for ≤ 24 months does not increase the risk of ECC. However, the evidence suggesting that longer‐duration breastfeeding increases risk was of low quality. The most recent meta‐analysis [12], which included nine case–control studies and 22 cohort studies, found no statistically significant risk of ECC in children breastfed beyond 12 months. The evidence generated by these reviews relied exclusively on primary studies published in English [7, 8, 9, 10, 11, 12]. One of the reviews also neglected significant portions of the gray literature [11], such as academic papers, theses, dissertations, research reports, conference papers, and ongoing research. These unjustified restrictions to English‐language or commercially published literature may have introduced publication bias [13].

Despite the aforementioned evidence, there is a lack of consensus regarding prolonged breastfeeding and the risk of ECC, particularly in the context of the WHO and UNICEF recommendations for breastfeeding up to 24 months or beyond. Additionally, there is a need to update the meta‐analysis with new evidence published since the last review, without imposing any language or database restrictions. Thus, the present SR aims to answer the following focused question: Does prolonged breastfeeding represent a risk for dental caries in children up to 71 months?

## 2 Material and Methods

The SR protocol was developed according to the PRISMA‐P (Preferred Reporting Items for Systematic Review and Meta‐Analyses protocols) [14] and registered in the International Prospective Register of Systematic Reviews (PROSPERO) under the number CRD42024509212. This SR was reported according to the PRISMA Statement (Preferred Reporting Items for Systematic Review and Meta‐Analyses checklist) (Appendix S1) [15].

### 2.1 Eligibility Criteria

The inclusion and exclusion criteria adopted in this study were structured following the acronym PECO (Population, Exposure, Comparison and Outcome), recommended for SRs [16]:

P—population: Children up to 71 months.

E—exposition: Prolonged breastfeeding > 12 months.

C—comparison: No comparison group or not breastfed at the same age.

O—Outcome: Risk of dental caries.

For inclusion, publications had to be observational studies published as full articles that compare the risk or prevalence of dental caries in children (up to 71 months) who have experienced prolonged breastfeeding (> 12 months) [17]. We did not restrict the date or language of publication.

The following exclusion criteria were considered: (1) children with known immunological diseases, with physical, neurological, or metabolic syndromes, with a chronic history of infection, or with a history of preterm birth (< 36 weeks); (2) children over 71 months old; (3) different types of feeding practices as the main outcome; (4) the absence of the comparison group or N/A; (5) dental hypoplasia and other dental abnormalities; (6) in vitro studies, animal studies, case reports, cross‐sectional studies, case–control studies, randomized trials, reviews, letters, personal opinions, book chapters, and conference abstracts.

### 2.2 Information Sources and Search Strategy

The search was performed up to May 17th and references were extracted from 8 main electronic databases: Pubmed (Medline), Scopus, EMBASE, Latin American and Caribbean Health Sciences (LILACS) (via BVS), Web of Science, Livivo, CINAHL (EBSCO), and Cochrane Library. In addition, the gray literature was searched on Google Scholar, Proquest, and Bibliografia Brasileira de Odontologia (BBO). No filters regarding language and date of publication were used. To identify the studies, terms referring to “Dental Caries,” and “Breastfeeding” were used, selected from the Health Sciences Descriptors (DeCS) and Medical Subject Headings (MeSH). Free terms were also selected. Strategies with specific word combinations and truncation were drawn up for each database with the support of a librarian specialized in health sciences (Appendix S2). We set up alerts for newly published articles containing our search terms. Additionally, we performed a hand search in the reference lists of published reviews to find articles that might have been missed during the platform search.

### 2.3 Selection Process

**FIGURE 1:**
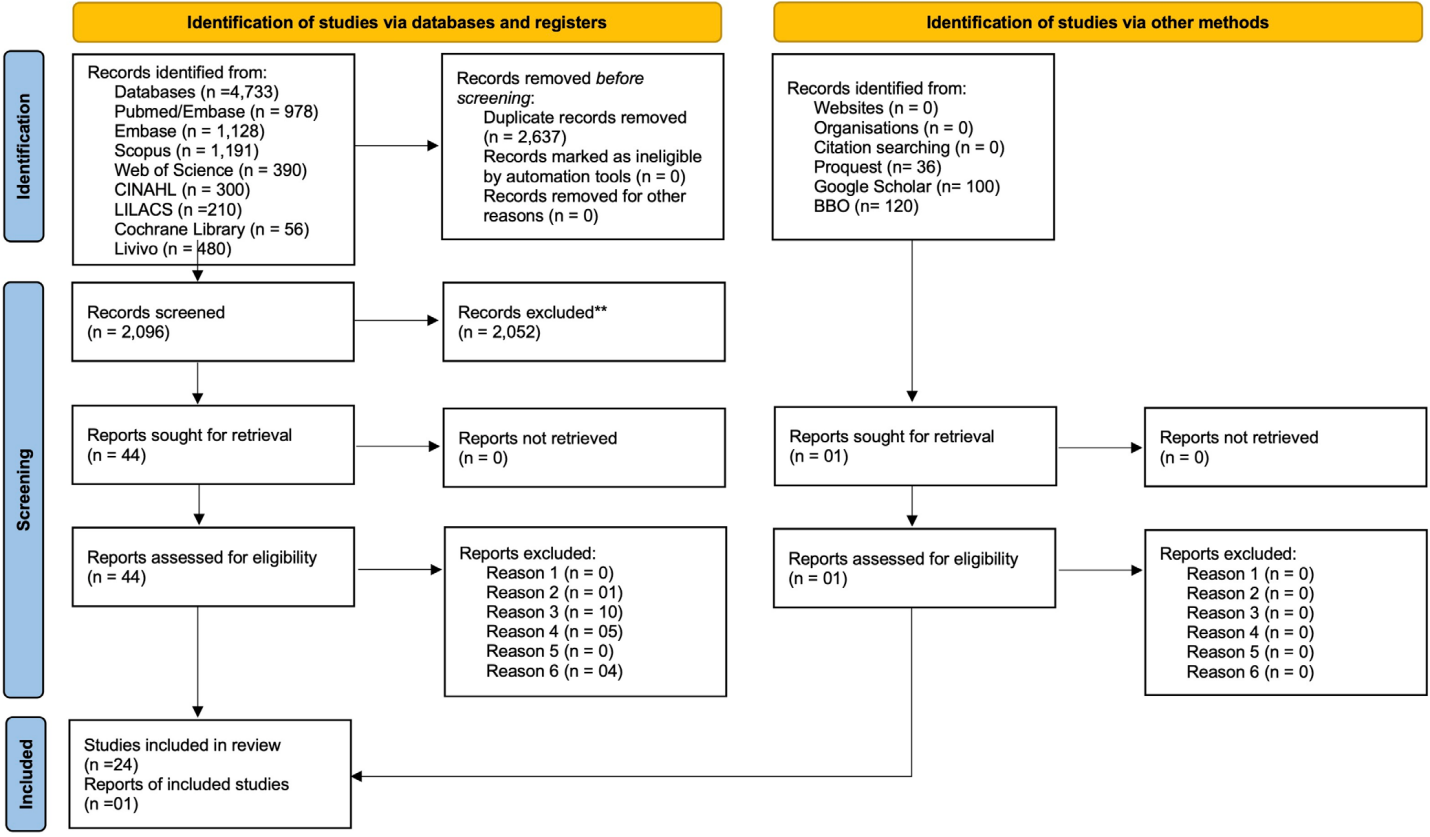
Flow chart showing the literature search process and selection criteria, adapted from the Preferred Reporting Items for Systematic Reviews and Meta‐Analyses guidelines (PRISMA, 2020, Adapted from Page et al. [15]). **Records excluded in Phase 1 based on title and abstract assessment.

Once the articles were identified in the specified databases, all duplicates were eliminated using the Endnote 20 digital application (EndNote version 21, The EndNote Team, Philadelphia, PA, USA). The selection of studies consisted of two phases. In the 1st phase, three reviewers (K.L., L.R.S.R., and R.M.R.), initially calibrated using the first 10 articles in the search, selected the studies by carefully reading the titles and abstracts using the Rayyan platform (Rayyan's web software, Qatar Computing Research Institute, Doha, Qatar). In situations of divergence, they were revisited by an expert reviewer (B.S.F.S.). In the 2nd phase, the same reviewers independently assessed the eligibility of the selected articles by reading the full texts. The conflicts were mediated by the same expert (B.S.F.S.). The complete selection process and reason for exclusion of the studies in Phase 2 are presented in Figure 1 and Appendix S3, respectively.

### 2.4 Data Collection Process

Information from the chosen primary studies was independently extracted by the same three reviewers from phases 1 and 2 using a designated data extraction form that included the following: identification (author, year of publication, country, and study design); sample characteristics (total number of participants divided into sex; follow up in years; age in months; breastfeeding duration; and bottle‐feeding duration); and the outcome (presence of caries; decayed, missing, filled teeth (DMFT/DMFS) index; nocturnal feeding; cariogenic diet; oral hygiene habits; breastfeeding frequency; bottle‐feeding frequency; and the results as well as their adjustment variables). The detailed information extracted from the selected studies is presented in Table 1. When the data required for qualitative and/or quantitative analysis was unavailable or incomplete or could not be derived from the reported values, attempts were made to obtain this information from the corresponding authors of the studies.

**TABLE 1:** Summary of descriptive characteristics of included articles.

Author, year	Country	Study design	Sample (*N*)	Gender (male/female)	Follow‐up	Age distribution (months)	Breastfeeding duration (< 12 months; 12–24 months; > 24 months)	Bottle feeding duration (< 12 months; 12–24 months; > 24 months)	Criteria for dental caries diagnosis	DMFT index^a^	Nocturnal feeding (yes or no)	Cariogenic diet (yes or no/description)	Oral hygiene habits (yes or no and/or description)	Breastfeeding frequency	Bottle feeding frequency	Risk or dental caries (RR/HR/OR 95% CI) and breastfeeding	Main conclusions
Abanto et al., 2022	Brazil	Cohort	800	—	2 years	—	< 12 months: 247 12–23 months: 439 ≥ 24 months: 114	No: 320 Yes: 480 2 years old^a^	WHO	—	—	No: 22 Yes: 778 1–3 times a day: 277 ≥ 4 times a day: 501	No: 25 Yes: 775 Toothbrushing Daily: 641 Weekly: 132	—	—	12–23 months: OR: 1.13 CI: 1.05–1.20 ≥ 24 months OR: 1.27 CI: 1.14‐1.40	Prolonged breastfeeding is a weak risk factor for dental caries. Reduced sugar consumption at 2 years old mitigates this risk. With a 22.8% early childhood caries rate, focused interventions are crucial in the first 1000 days to promote healthy feeding, breastfeeding, and limited sugar intake
Barroso et al., 2021	Brazil	Cohort	132	Male: 62 Female: 70	3 years	< 24 months: 83 24–36 months: 49	< 24 months: 66 > 24 months: 66	< 24 months: 81 > 24 months: 51	ICDAS	—	No	Yes Low sugar consumption: 70 High sugar consumption: 62	Supervised Toothbrushing: 108 Not Supervised Toothbrushing: 24	—	—	> 24 months: RR 2.24 CI:1.23–4.08	In conclusion, breastfeeding for more than 24 months was a risk factor for severe dental caries, alongside early childhood caries and non‐nuclear family structure. Caution is needed when extrapolating these results due to uncontrolled variables like breastfeeding frequency and nighttime breastfeeding habits
Bernabé et al., 2016	Scotland	Cohort	1.102	Male: 592 Female: 510	4 years	Birth‐48 months	< 6 months: no related > 6 months: 194	—	—	—	—	—	—	—	—	Coefficient (linear mixed effects) < 6 months: 0.02 CI: −0.23‐0.28 > 6 months: 0.06 CI: −0.25‐0.37	This prospective study reveals that low birth weight and maternal smoking, rather than breastfeeding duration, were linked to caries progression from ages 1 to 4 in Scottish children
Birungi et al., 2017	Uganda	Cohort	417	Male: 208 Female: 209	5 years	Birth‐60 months	< 24 months: 196 > 24 months: 188	—	WHO	DMFT	—	Yes: Family sugar consumption index Less: 16% More 84%	Family oral hygiene index: Bad: 508 Good: 1577	—	—	Exclusive breastfeeding: IRR 0.60 CI: 0.41–0.88 > 24 months IRR 1.01 CI: 0.97–1.04	The study suggests that Directed Acyclic Graphs (DAGs) aided in assessing the causal impact of Exclusive Breastfeeding (EBF) on Early Childhood Caries (ECC) among 5‐year‐old Ugandan preschoolers. Results indicate a protective effect of 24 weeks of EBF on ECC. Further research, incorporating unmeasured variables from the DAGs, is crucial for robust causal assertions
Blanco et al., 2021	Spain	Cohort	335	Male: 189 Female: 146	> 12 months	48–60 months: 335	< 12 months: 254 > 12 months: 81	—	—	—	—	Yes: Sugar ingestion (mg/day) Mean ingestion sugar a day: about: 110,58	—	—	—	1–3 months: Crude OR 0.93 CI: 0.25–3.4 3–6 months: Crude OR 0.76 CI: 0.25–2.32 6–9 months: Crude OR 0.38 CI: 0.07–1.93 9–12 months: Crude OR 1.97 CI: 0.67–5.78 > 12 months: Crude OR 2.6 CI: 1.02–6.61	Breastfeeding for over 12 months correlates significantly with higher Early Childhood Caries (ECC) prevalence, while breastfeeding up to 12 months acts protectively against ECC. More research, incorporating fluoride toothpaste hygiene, is needed to explore this association further. No direct link was found between total energy or sugar intake and ECC prevalence
Chaffee et al., 2014	Brazil	Cohort	715	—	38 months	36–48 months (715)	< 12 months: 468 12–23: 65 > 24 months: 156	—	WHO	DMFS 3.2	—	Consuming sweet substances in bottle at age 5–9 months: 198 Consuming sweet substances in bottle at age 2–3 years: 312	—	—	—	6–11 months: PR 1.77 CI: 1.12–2.85 12–23 months: PR 1.82 CI: 0.85–3.20 24 or more months: PR 2.10 CI: 1.50–3.25	Finally, this study population, featuring a relatively high prevalence of breastfeeding and of caries, might not be representative of the breastfeeding‐caries relationship in all historical, geographical, and socioeconomic contexts
Devenish et al., 2020	Australia	Cohort	965	—	1 year	24–36 months	0‐1 months: 94 1–6 months: 257 6 to 12 months: 228 ≥ 12 months: 386	—	WHO	—	—	Yes: WHO guideline for sugars intake: Noncompliant: 134 Partially noncompliant: 224 Semi‐compliant: 326 Compliant: 281	—	—	—	0 to < 1 month: PR 0.64. CI: 0.25–1.62 1 to < 6 months: PR 0.85 CI: 0.46–1.56 6 to 12 months: PR 1.00 > 12 months: PR 1.42 CI: 0.85–2.38	The study found no connection between breastfeeding until at least 1 year of age, including nighttime breastfeeding, and early childhood caries by ages 2 to 3. It emphasizes the importance of promoting breastfeeding initiation and duration in accordance with global and national recommendations for overall health improvement
Feldens et al., 2010.	Brazil	Cohort	340	—	4 years	48 months: 340	< 12 months: 164 > 12 months: 176	—	WHO	—	12 months: 197	Yes: 91	Yes, Toothbrushing with fluoride paste: 285	0–2: 192 3–6: 31 > 7: 117	—	All 12 months: 0–2 daily: Crude RR 1.00 3–6 daily: Crude RR 2.04 CI: 1.22–3.39 7 or more daily: Crude RR 1.97 CI: 1.45–2.68	In conclusion, targeting early dietary factors associated with Severe Early Childhood Caries (S‐ECC), programs for infants and toddlers should stress reducing high‐sugar foods and maintaining meal intervals. Further research is urged to develop strategies for complex feeding practices, while emphasizing maternal education to combat childhood diseases
Feldens et al., 2018	Brazil	Cohort	345	—	38 months	38 months: 345	at 12 months: 174	—	WHO	D1MFT: 345	—	Yes: Sugar introduction before age 6 months: 344	—	—	—	All 12 months: Moderate/high frequency mixed‐feeding (both 1 or more/day): RR 1.45 CI: 1.02–2.07 High‐frequency bottle‐use only (> 3/day): RR 1.37 CI: 0.98–1.92 High‐frequency breastfeeding only (> 3/day): RR 1.82 CI: 1.28–2.57	In this population, a correlation was found between feeding frequency at 12 months—including breastfeeding and bottle use—and dental caries status at age 3. Preventive measures could include promoting less frequent nursing once complementary foods are introduced and limiting snack and drink frequency. Feeding advice should consider both nutritional needs and caregivers' beliefs
Helderman et al., 2006.	Myanmar	Cohort	163	Male: 84 Female: 79	3 years	25 to < 27 months: 41 > 27 to 30 months: 122	Low 50%: 81 High 50%: 82	—	—	—	Breast nipple in child's mouth at night: No: 122 Yes: 41	—	Toothbrushing before the age of 18 moths: No 147 Yes: 16	—	—	All 12 months: > 4 daytime breastfeeding: Crude OR 4.9 CI: 0.9 25.3 > 15 min/breastfeeding (day): Crude OR 3.9 CI: 0.8–19.1	The present study indicates that, besides the known caries‐inducing factors such as consumption of sugars, the consumption of pre‐chewed rice and nocturnal breastfeeding after the age of 12 months pose a risk for the child's developing ECC in this Myanmar community
Hong et al., 2014.	United States	Cohort	509	—	9 years	—	6 months: 361 6–12 months: 92 > 12 months: 56	—	WHO	—	—	—	Yes, Daily fluoride intake Tooth‐brushing	—	—	Prevalence: < 6 months: 25% > 6 months: 19%	Continuing breastfeeding beyond 6 months appears to lower the risk of early childhood caries in the early years. Further research with comprehensive data on breastfeeding patterns, duration, quality, and quantity, along with early‐life caries examination data, is needed
Ibrahim et al., 2009.	Osaka, Japan	Cohort	283	—	3.5 years	36–48 months: 283	—	—	—	—	—	—	—	—	—	18 months: Crude OR 4.7 CI: 0.9–24 30 months: Crude OR 2.4 CI: 0.6–9.2	The Cariostat score at 1.5 years not only reflects current oral health but also predicts oral condition at 2.5 and 3.5 years. Similarly, the score at 2.5 years demonstrates this predictive ability. Children's lifestyle evolves with age, potentially impacting their caries risk status
Ji et al., 2006	Japan	Cohort	392	—	3.5 years	36–48 months: 392	—	—	—	—	—	—	Yes, Supervised Toothbrushing	—	—	18 months: Crude OR 3.26 CI: 1.60–6.62 30 months: Crude OR 5.54 CI: 0.97–31.67	Breast feeding was the most influential factor. Actually in this study, breast feeding was the preferred with time of infant feeding before sleeping or during midnight. We found that 18‐month‐old children who breast‐fed during waking hours oral ready weaned compared with breast feeding children, are not likely to be at higher risk for caries experience when the children was 42‐month‐old
Lunteren et al., 2020	Netherlands	Cohort	4146	—	6 years	—	Breastfeeding duration: 0–6 months: 1980 6–12 months: 850 > 12 months: 386	—	—	DMFT = 0 *n* = 2988 DMFT > 0: *n* = 1158	Never: 2832 Ever: 409 Missing data: 905	Intake of sugar‐containing products at 6 years Low (≤ 2 times per day):1407 High: (> 2 times per day): 2739	—	—	—	0–6 months: OR 1.00 6–12 months: OR 1.13 CI: 0.92–1.38 > 12 months: OR 1.35 (CI: 1.04–1.74)	We have shown that prolonged breastfeeding as well as bottle‐feeding during the night are associated with an increased risk of childhood dental caries. Our findings confirm the results of earlier studies in other countries and add that the associations are independent of SEP and sugar intake, and also exist in a European context that is without water fluoridation. Although future studies are encouraged that will study the potential mechanism be‐tween prolonged breastfeeding and dental caries in more detail, the evidence so far clearly shows a higher risk of dental caries in children being breastfed for > 12 months
Mathias et al., 2023	Brazil	Cohort	3645	Male: 1840 Female: 1805	4 years	36–48 months	< 24 months: 2600 > 24 months: 968	—	—	—	—	Yes UPF: Low/medium: 2591 High: 986	Yes, Frequency of toothbrushing: < 2 times a day: 1046 > 2 times a day: 2529	—	—	< 24 months breastfeeding: RR 1.00 > 24 months breastfeeding: RR 2.47 CI: 1.97–3.10	In conclusion, this study found no interaction between breastfeeding and UPF consumption, showing that the two exposures have different role on risk of ECC. The findings of the present study reinforce the need to adopt effective strategies to reduce the consumption of UPF and sugar‐sweetened beverages in the early stages of life, and pediatric dentists should consider the potential caries risk of breastfeeding for 24 months or beyond
Nakamura, 2009.	Brazil	Doctoral thesis	135	—	2 years and 11 months	< 35 months	—	—	WHO	—	—	—	—	—	—	OR: 31.1136 CI: 4.50–215.15	Incidence of ECC in babies indicate a strong polarization of dental caries in this sample, whose elements showed high susceptibility to caries, characterized by a high incidence in a short period after eruption, with the largest number of lesions occurred in the period corresponding to the infectivity window
Nirunsittirat et al., 2016	Thailand	Cohort	556	Female: 276 Male: 280	3–4 years	36–48 months	< 6 months: 212 6–11 months: 180 12–17 months: 156 > months: 8	—	WHO	WHO	—	Sweet consumption: < 3 times/week: 33 4–6 times/week: 249 7–9 times; week: 198 > 10 times/week: 76	Brushing frequency: None: 5 Not every day: 256 Every day: 240	Full breastfeeding: < 6 months: 212 6–11: 180 12–17: 156 > 18 months: 8	—	< 6 months: RR 1.0 6–11 months: RR 0.77 CI: 0.63,0.93 12–17 months: RR 0.93 CI: 0.77–1.12 > 18 months: RR 0.67 CI: 0.40–1.11	In conclusion, this prospective study suggests the benefit of full breastfeeding for 6–11 months for dental caries prevention in primary teeth. There was no association be‐tween the duration of any breastfeeding and dental caries. Prolonged breastfeeding was not associated with dental caries in this population
Nunes et al., 2012	Brazil	Cohort	260	—	—	18–42 months	—	—	WHO	DMFT 0.8 (+/−1.7)	Bottle‐feeding: 0 = 186 1 = 48 2 or more times = 7	Daily sucrose consumption between main meals 0 = 24 1 = 45 2 = 76 3 or more times = 96	—	—	—	IRR 1.15 CI: 0.84–1.59	The present results showed that prolonged breast‐feeding was not a risk factor for ECC after adjustment for a handful of important confounders. Age, sucrose consumption between main meals, and quality of oral hygiene were associated with ECC in a low‐income population, using a hierarchical analysis
Peres et al., 2017	Brazil	Cohort	1128	—	4 years	36–48 months	0–12 months: 741 13–23 months: 129 > 24 months: 258	—	—	DMFS: 4.05; SD: 7.38	—	—	—	—	—	Mean ratio: > 12 months: 1.0 13–23 months: 0.9 CI: 0.6–1.3 > 24 months or beyond: 1.9 CI: 1.5–2.4	Breastfeeding for ≥ 24 months increases the risk of having S‐ECC. We suggest adopting measures to prevent dental caries in childhood as early as possible, because breastfeeding is beneficial for children's health
Pires et al., 2020	Brazil	Retrospective cohort	310	Male: 159 Female: 151	—	0–36 months	< 9 months: 159 > 9 months: 151	—	—	DMFT index: ≤ 2: 165 > 2: 145	Yes: 196 No: 114	Yes: 219 No: 84	Toothbrushing frequency: Once a day: 105 Two times a day or more: 76	—	—	≤ 9 months: OR 1.00 ≥ 9 months: OR 0.38 CI: 0.21–0.68	A higher caries experience in early childhood is not associated to child's daytime caring person. On the other hand, the higher caries experience is associated with low caregiver schooling and older children
Sæthre, Wang & Wigen, 2023.	Norway	Cohort	1088	Male: 583 Female: 505	5 years	—	Stop 6 months: 241 Stop 8 months: 167 Stop: 11 months: 241 Stop: 14 months: 238 Stop 18 months: 174	—	—	—	Breastmilk: 64 Sugary drink or milk: 113	Less than once a week 529 Once a week or more often: 525	Twice daily: 588 Less than twice daily: 527	—	—	6 months: OR 1.3 CI: 0.5–3.1 8 months: OR 1.4 CI: 0.6–3.5 11 months: OR 0.8 CI: 0.3–2.1 14 months: OR 1.3 CI: 0.5–3.3 18 months: OR 1.00	There was no association between breastfeeding up to 18 months of age and caries development during preschool age. Caries prevalence at 5 years of age was associated with high frequency of sugar intake and a low frequency of tooth brushing with fluoride toothpaste
Sritangsirikul et al., 2024	Thailand	Cohort	486	Male: 244 Female: 242	2 years	0–36 months	Duration of full breastfeeding < 6 months: 219 6–11 months: 166 12–17 months: 75 ≥ 18 months: 26 Duration of any breastfeeding < 6 months: 205 6–11 months: 129 12–17 months: 64 ≥ 18 months: 88	—	WHO	—	Never: < 6 months: 60 6–11 months: 64 12–17 months: 23 ≥ 18 months: 5 1–3 times/week: < 6 months: 46 6–11 months: 38 12–17 months: 14 ≥ 18 months: 3 > 3 times/week: < 6 months: 113 6–11 months: 64 12–17 months: 38 ≥ 18 months: 18	Number of meals: 1–2 meals: < 6 months: 84 6–11 months: 56 12–17 months: 16 ≥ 18 months: 2 ≥ 3 meals: < 6 months: 135 6–11 months: 110 12–17 months: 59 ≥ 18 months: 24 Sugar consumption between meals: ≤ 3 times/week: < 6 months: 78 6–11 months: 53 12–17 months: 22 ≥ 18 months: 8 4–6 times/week: < 6 months: 51 6–11 months: 47 12–17 months: 21 ≥ 18 months: 9 ≥ 7 times/week: < 6 months: 90 6–11 months: 66 12–17 months: 32 ≥ 18 months: 9	Toothbrushing: Never < 6 months: 42 6–11 months: 18 12–17 months: 11 ≥ 18 months: 4 < 2 times/day: < 6 months: 90 6–11 months: 63 12–17 months: 28 ≥ 18 months: 4 ≥ 2 times/day: < 6 months: 87 6–11 months: 85 12–17 months: 36 ≥ 18 months: 18 Fluoride toothpaste—Yes: < 6 months: 44 6–11 months: 40 12–17 months: 20 ≥ 18 months: 11 Fluoride toothpaste—No: < 6 months: 175 6–11 months: 126 12–17 months: 55 ≥ 18 months: 15	—	—	Full breastfeeding: < 6 months: RR 1.0 6–11 months: RR 0.78 CI: 0.64–0.94 12–17 months: RR 0.79 CI: 0.62–1.02 ≥ 18 months: RR 0.94 CI: 0.77–1.13 Any breastfeeding: < 6 months: RR 1.0 6–11 months: RR 0.93 CI: 0.77–1.12 12–17 months: RR 1.1 CI: 0.85–1.43 ≥ 18 months: RR. 1.45 CI: 1.31–1.60	In conclusion, our findings support the value of continued breastfeeding throughout the first year of life and beyond, as recommended by leading organizations. A longer duration of full breastfeeding can protect against early childhood caries. However, any breastfeeding (with or without formula milk) for ≥ 18 months increases the caries prevalence. Therefore, breastfeeding practices should be strongly encouraged, along with urging caregivers to provide proper oral hygiene and dietary practices for children
Tada et al., 1999	Japan	Cohort	392	Male: 215 Female: 177	3 years	24–36 months: 392	—	—	WHO	DMFT All teeth 18 months: 0.21 ± 0.87 36 months: 1.81 ± 3.12	—	Sweet foods No: 259 Yes: 133 Sweet beverages No: 139 Yes: 253	Frequency of toothbrusing: Under once/day: 94 Once/day: 222 Over/twice day: 74	—	—	18 months: OR 1.00 36 months: OR 6.65 CI: 2.89–15.20	In conclusion, our data on dental caries occurrence and caries risk factors of infants indicate that bottle feeding and breast feeding were related to the increment of the DMFT from 18 months of age to 3 years old. This calls for dental health education with in‐ struction on milk feeding before the 18‐month‐old check‐up
Tashiro et al. 2021	Japan	Cohort	387	Male: 200 Female: 187	2 years	24–36 months	18 months: Yes: 110 No: 231	18 months Yes: 35 No: 306	—	—	—	Sweet beverage intake ≥ 4 times/week: 102 < 4 times/week: 238	Frequency of tooth brushing by parents ≥ twice/day: 96 < twice/day: 245	—	—	18 months: OR: 7.106 CI: 2.857–19.455	In conclusion, the present results showed that prolonged breastfeeding was a risk factor for ECC at 18 months of age after adjustment for a number of important confounding factors. A close association was also demonstrated between quality of oral hygiene and ECC at 18 months and 3 years of age. Children brought in for regular examination and consultation under the oral care program from 12 months of age were less likely to develop ECC at 18 months and 3 years of age
Yokoi et al. 2021	Japan	Cohort	640	—	18 months	24–36 months: 640	> 18 months: 178	> 18 months: 66	WHO	—	—	3 times a day = 126	Yes: 537 No: 103	—	—	> 18 months: OR 1.71 CI: 1.15–2.55	In conclusion, receiving daytime care at a nursery school, prolonged breastfeeding, and a high frequency of snacking were significantly associated with ECC risk in Japanese toddlers

Abbreviations: —, not reported; CI, confidence interval; IRR, incidence rate ratio; OD, odds ratio; PR, prevalence ratio; RR, risk ratio; WHO, World Health Organization.

^a^ Decay‐missing‐filled teeth index.

### 2.5 Risk of Bias Assessment (Methodologic Quality)

The methodologic quality analysis of the included studies was independently performed by three reviewers (K.L., L.R.S.R., and R.M.R.), initially calibrated using five included studies, using the Joanna Briggs Institute Critical Appraisal Checklist for cohort studies [18]. An expert (B.S.F.S.) was consulted in case of disagreement between the three reviewers. Eleven items were answered with “yes,” “no,” “unclear,” or “not applicable” for each article. The risk of bias was assessed and reported separately for each study examined and was categorized as “high,” “moderate,” and “low” when the study achieved a “yes” score of 49%, 50% to 69%, and 70%, respectively. The total not applicable (NA) items were excluded from the sum. This scoring system was adopted as previously described to simplify the classification of the risk of bias [19].

### 2.6 Effect Measures

The main outcome was the association between prolonged breastfeeding and the incidence/prevalence of ECC. For data extraction, we sought to retrieve adjusted measures (relative risk (RR), odds ratio (OR), prevalence ratio (PR), and others) when they were available. In case those were not available, crude measures were extracted for data summarization.

### 2.7 Synthesis Methods

The software Review Manager (version 5.3) was used for the meta‐analysis (*Review Manager—RevMan*, Version 5.4; The Cochrane Collaboration, 2020. Available online: https://revman.cochrane.org—accessed on 15 June 2024). Heterogeneity among the studies was assessed using Cochran's *Q* test and the *I* ^2^ index. An *I* ^2^ index of 0%–30% indicated insignificant heterogeneity, 30%–50% indicated moderate heterogeneity, 50%–90% indicated substantial heterogeneity, and above 90% indicated considerable heterogeneity. Depending on the degree of heterogeneity, either fixed‐effect or random‐effects models were employed to combine the data [20].

Sensitivity analysis was performed excluding one study at a time to screen for studies that might overly estimate or underestimate results. Additionally, a subgroup analysis was planned to separate the studies according to the outcome measures used, considering whether they were measures of incidence or prevalence and whether the data were adjusted or crude. Subgroup analyses considering nocturnal breastfeeding were not performed due to the lack of information on this practice in the studies. The absence of reporting does not necessarily indicate that nocturnal breastfeeding was absent in the sample. All statistical analyses were performed at a predetermined significance level of 0.05 and 95% CIs.

### 2.8 Bias of Publication Assessment

To assess publication bias, funnel plots of the log OR/RR against their standard errors were created to visually examine funnel plot asymmetry, which indicates the presence of publication bias [20, 21, 22, 23]. A significance level of *p* < 0.05 was applied. The software Review Manager was used for data entry and analysis.

### 2.9 Certainty Assessment

We evaluated the certainty of evidence using the Grades of Recommendation, Assessment, Development, and Evaluation (GRADE) system. This tool comprises five domains: risk of bias, inconsistency, indirectness, imprecision, and publication bias. The certainty level for the body of evidence is categorized as high, moderate, low, or very low. The summary of findings table was produced using the Grading of Recommendations Assessment, Development and Evaluation online software (GRADEpro GTD) [24].

## 3 Results

### 3.1 Study Selection

In Phase 1, a search in eight main electronic databases yielded 4733 references. After removing duplicates, 2095 references remained for the title and abstract assessment. In Phase 2, 44 studies underwent a full‐text comprehensive appraisal. Applying the eligibility criteria, 24 studies remained for further analysis. An additional search in the gray literature yielded 1 more study. Further hand searches in the reference lists of the 25 included articles did not yield any additional studies. The specific reasons for exclusion in Phase 2 are presented in Appendix S3. The detailed selection process is illustrated in the selection process flow diagram in Figure 1.

### 3.2 Study Characteristics

**FIGURE 2:**
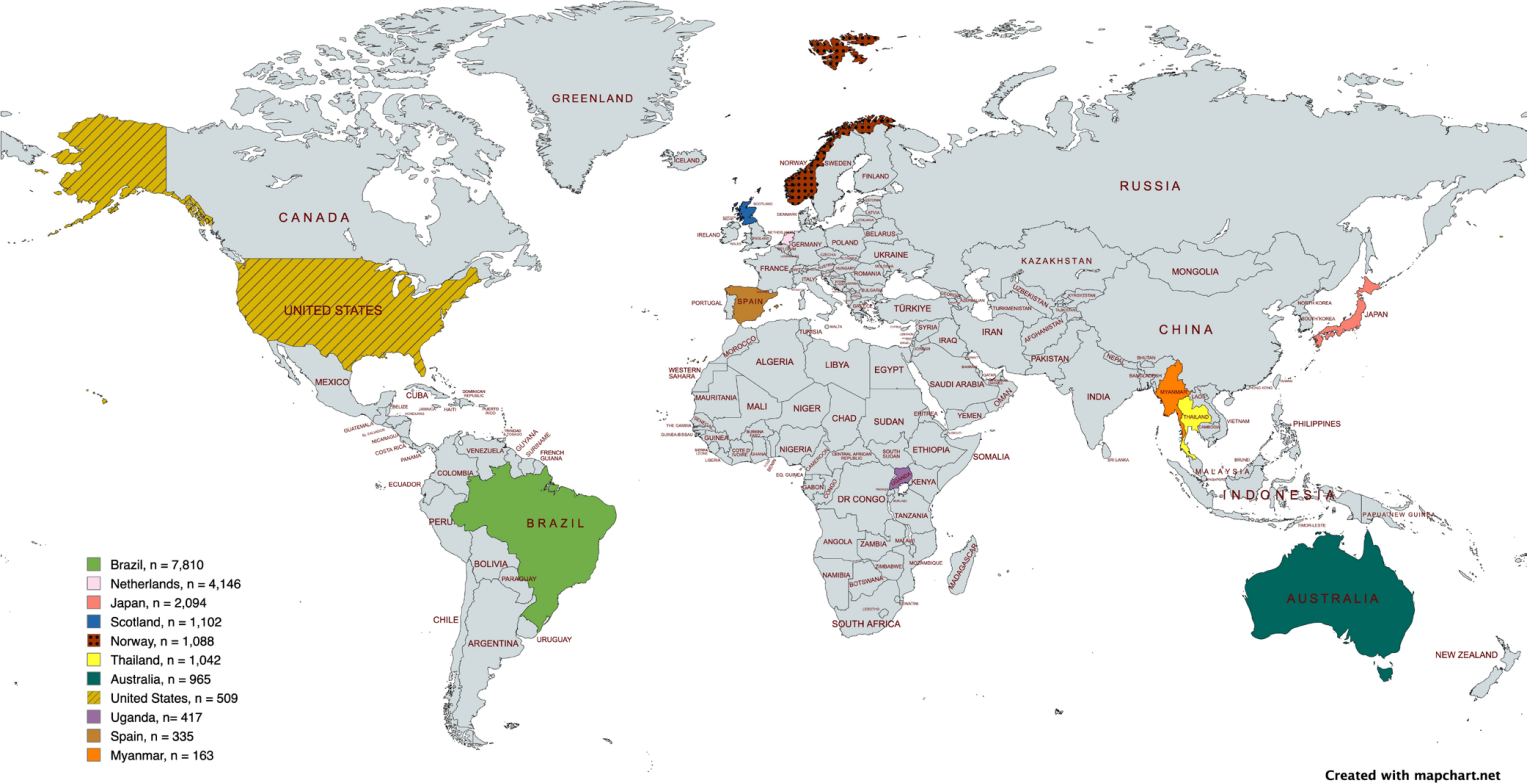
Geographical distribution of the sample and its representation in the pooled sample.

Out of the 25 selected studies, eight presented incidence data on the risk of caries in prolonged breastfeeding [25, 26, 27, 28, 29, 30, 31, 32], and 17 provided data in terms of odds ratio [33, 34, 35, 36, 37, 38, 39, 40, 41, 42, 43, 44, 45, 46, 47, 48, 49]. Most studies were from Brazil [25, 28, 29, 30, 33, 36, 43, 44, 45], followed by Japan [40, 41, 47, 48, 49], Thailand [31, 32], and other countries represented by one study each: Scotland [34], Uganda [27], Spain [35], Australia [37], Myanmar [38], the United States [39], the Netherlands [42], and Norway [46]. The complete geographical distribution of the sample and its representation in the pooled sample are illustrated in Figure 2. These cohorts were published between 1999 and 2024, with sample sizes ranging from 132 to 4146 participants. The pooled sample included 19 681 participants. Twelve of the 25 selected studies provided the male‐to‐female proportion, resulting in a pooled proportion of 1.07:1. Most of the cohort studies reported follow‐up periods ranging from 1 to 9 years.

The main criteria adopted in the studies for diagnosing dental caries was the WHO criteria [27, 28, 29, 30, 31, 32, 33, 36, 37, 39, 43, 47, 49]. One study adopted the International Caries Detection and Assessment System (ICDAS) [26], and 11 studies did not report the criteria adopted [25, 34, 35, 38, 40, 41, 42, 44, 45, 46, 48]. Nocturnal feeding was reported in seven studies [28, 30, 32, 38, 42, 45, 46], with a heterogeneous mix of breastmilk, bottle milk, and sugary drinks. The cariogenic diet was assessed through the consumption of sugary products [26, 27, 28, 29, 31, 33, 36, 42, 45, 46] or ultra‐processed foods [25]. The frequency of cariogenic diet consumption was assessed in some studies [27, 31, 32, 35, 37, 42], and the quantity in other [35]. However, seven studies did not report the consumption of cariogenic diet [34, 38, 39, 40, 43, 44]. Regarding oral hygiene habits, the main reports included the frequency of toothbrushing [25, 31, 32, 33, 39, 45, 46, 47, 48], and whether it was supervised [26, 48]. Some studies only reported the presence or absence of oral hygiene habits [28, 38], one analyzed the family oral hygiene index [27], while others did not report these habits at all [29, 30, 34, 35, 36, 37, 40, 42, 43, 44].

### 3.3 Risk of Bias Within Studies

**FIGURE 3:**
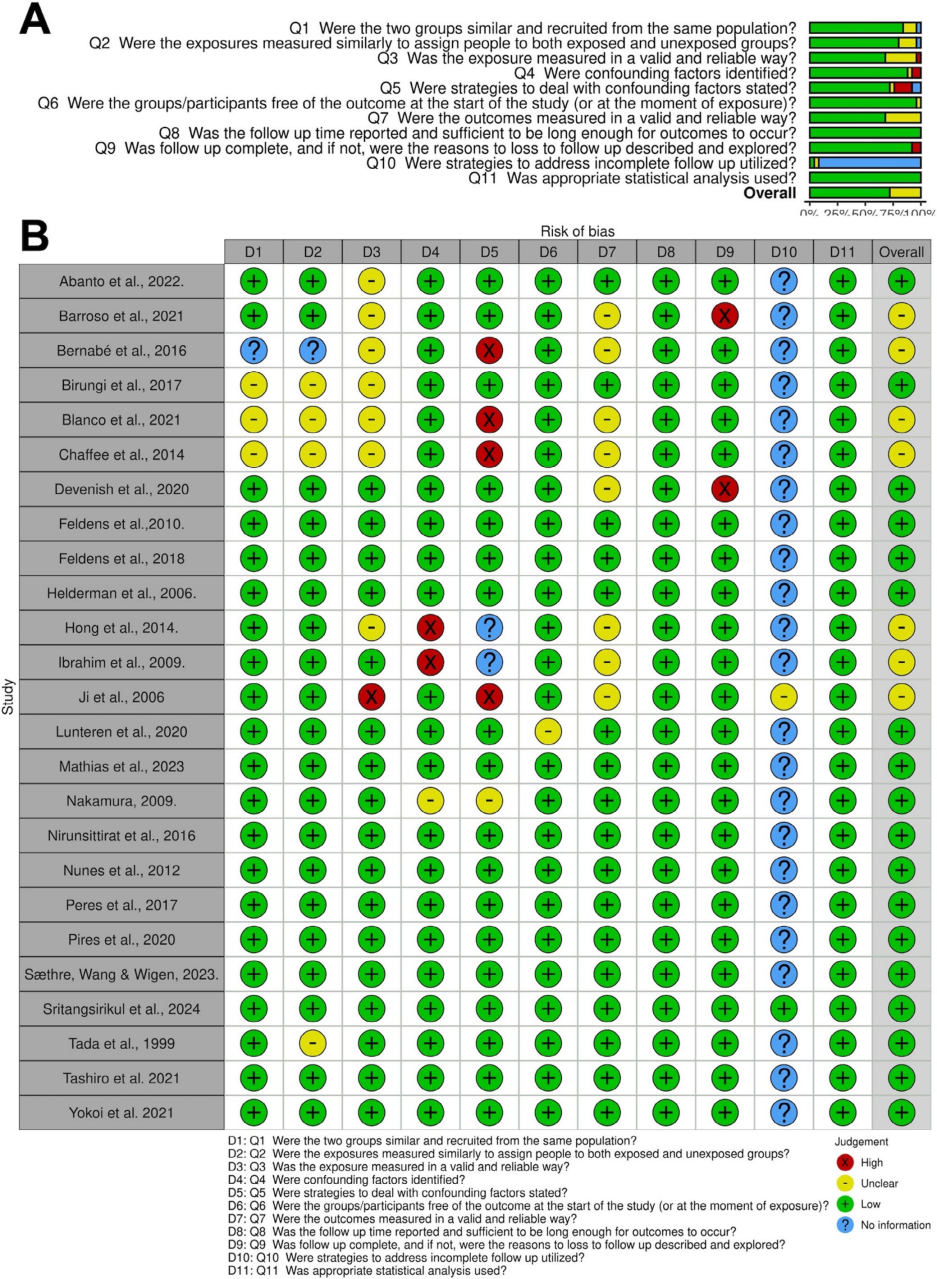
Risk of bias analysis of each cohort study included, using the Joanna Briggs Institute Critical Appraisal Checklist for cohort studies.

Within the selected cohort studies, a primary concern regarding bias was whether strategies to manage confounding factors were explicitly stated. Some studies did not describe how they accounted for cariogenic diets and oral hygiene habits affecting the risk of ECC in prolonged breastfeeding samples [33, 34, 35, 36]. Additionally, the question about strategies for dealing with incomplete follow‐up was often deemed high, as all studies had no complete participant follow‐up. Overall, most studies were rated as having a “Low,” with 4 cohorts classified as “Moderate,” and none classified as “High.” Detailed bias assessments are presented in Figure 3 and Appendix S4.

### 3.4 Synthesis of Results

Some primary studies did not provide the necessary raw data to construct contingency tables for calculating OR or RR. Consequently, separate meta‐analyses were conducted using random effects models. For subgroup analysis, breastfeeding durations of 12–24 months and over 24 months were the most frequently reported categories in the studies.

**FIGURE 4:**
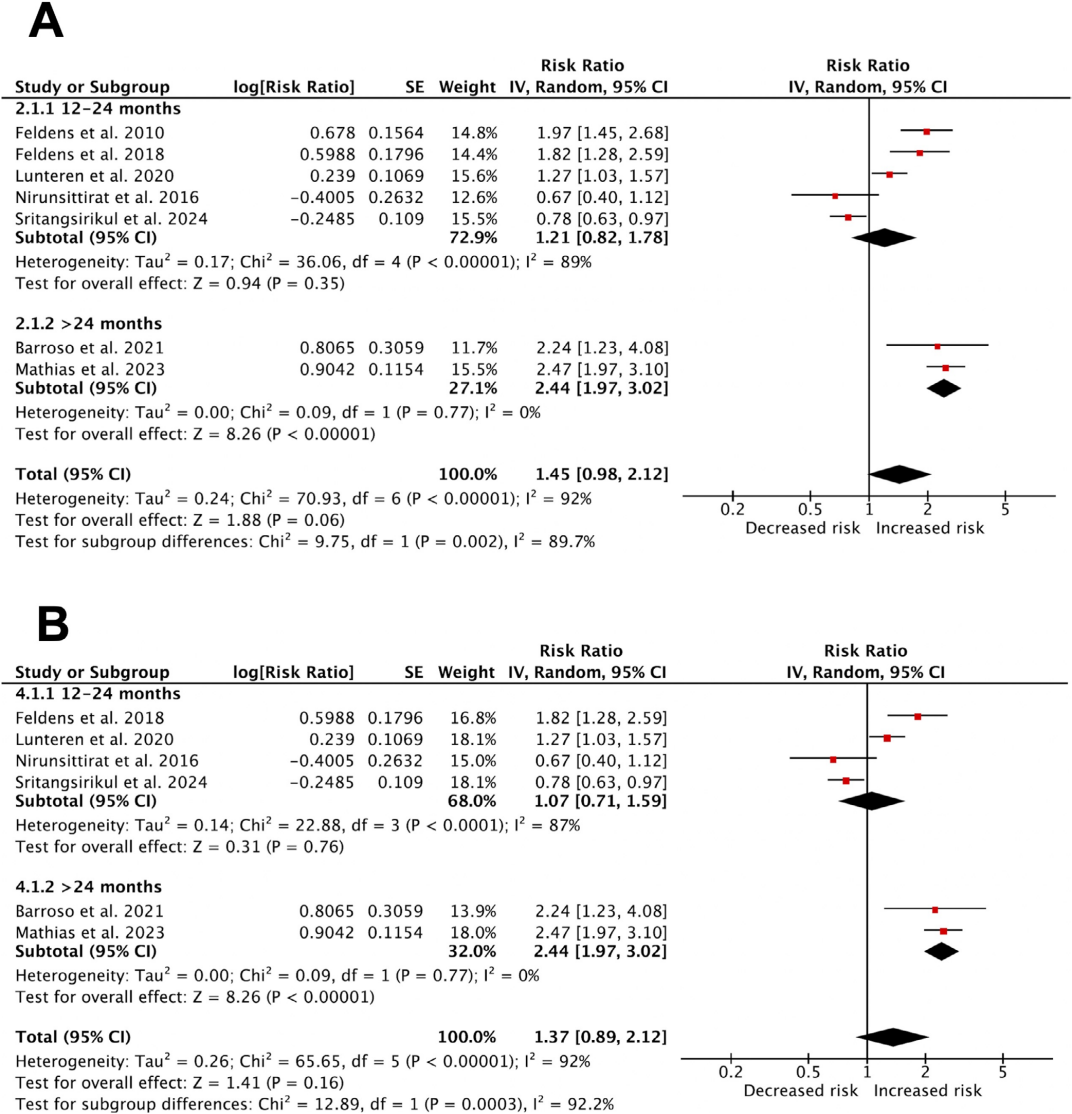
Forest plot in (A) displaying the overall relative risk (RR) of Early Childhood Caries (ECC) in children exposed to prolonged breastfeeding (> 12 months). In (B), the overall RR of ECC associated with breastfeeding exceeding 12 months highlights the results of the sensitivity analysis.

The risk of ECC was significantly higher in children breastfed for more than 24 months (RR = 2.44; 95% CI, 1.97 to 3.02; *I* ^2^ = 0%; *p* = 0.77; Figure 4A), based on pooled evidence from two cohorts. For the 12–24 month period, there was no significant increase in ECC risk (RR = 1.21; 95% CI, 0.82 to 1.78; *I* ^2^ = 89%; *p* = 0.00001; Figure 4A). Sensitivity analysis highlighted the influence of the study by Feldens et al. [28] on the overall risk of ECC associated with breastfeeding exceeding 12 months (Figure 4B).

**FIGURE 5:**
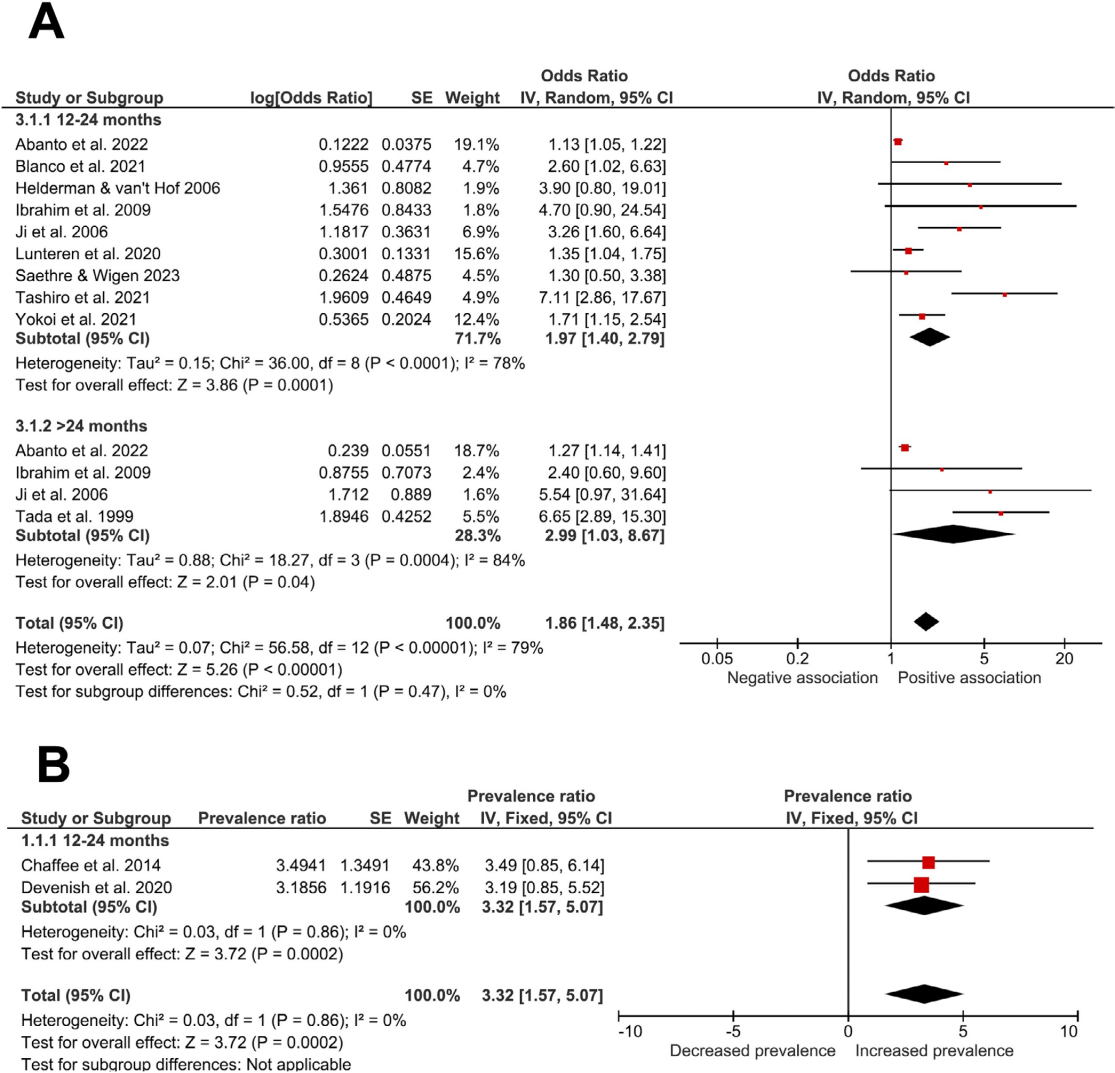
Forest plot presenting the prevalence of ECC in children exposed to prolonged breastfeeding (> 12 months). In (A), the data are presented in odds ratio (OR), and in (B), they are presented in prevalence ratio (PR).

Meta‐analyses indicated an overall higher prevalence of ECC in children breastfed for > 12 months (OR = 1.86; 95% CI, 1.48 to 2.35; *I* ^2^ = 79%; *p* = 0.00001; Figure 5A), and a notably increased prevalence for those breastfed beyond 24 months (OR = 2.99; 95% CI, 1.03 to 8.67; *I* ^2^ = 84%; *p* = 0.0004; Figure 5A). Regarding PR, pooled data from 2 cohorts showed an increased ECC prevalence between 12 and 24 months of breastfeeding (PR = 3.32; 95% CI, 1.57 to 5.07; *I* ^2^ = 0%; *p* = 0.0002; Figure 5B).

Five studies were excluded from the meta‐analysis for specific reasons: three lacked data on children older than 12 months despite follow‐up exceeding 12 months [34, 39, 45]; one did not clearly distinguish breastfeeding durations of 12–24 months from over 24 months [43]; and one utilized “mean ratio” as the effect measure [44].

### 3.5 Reporting Biases

**FIGURE 6:**
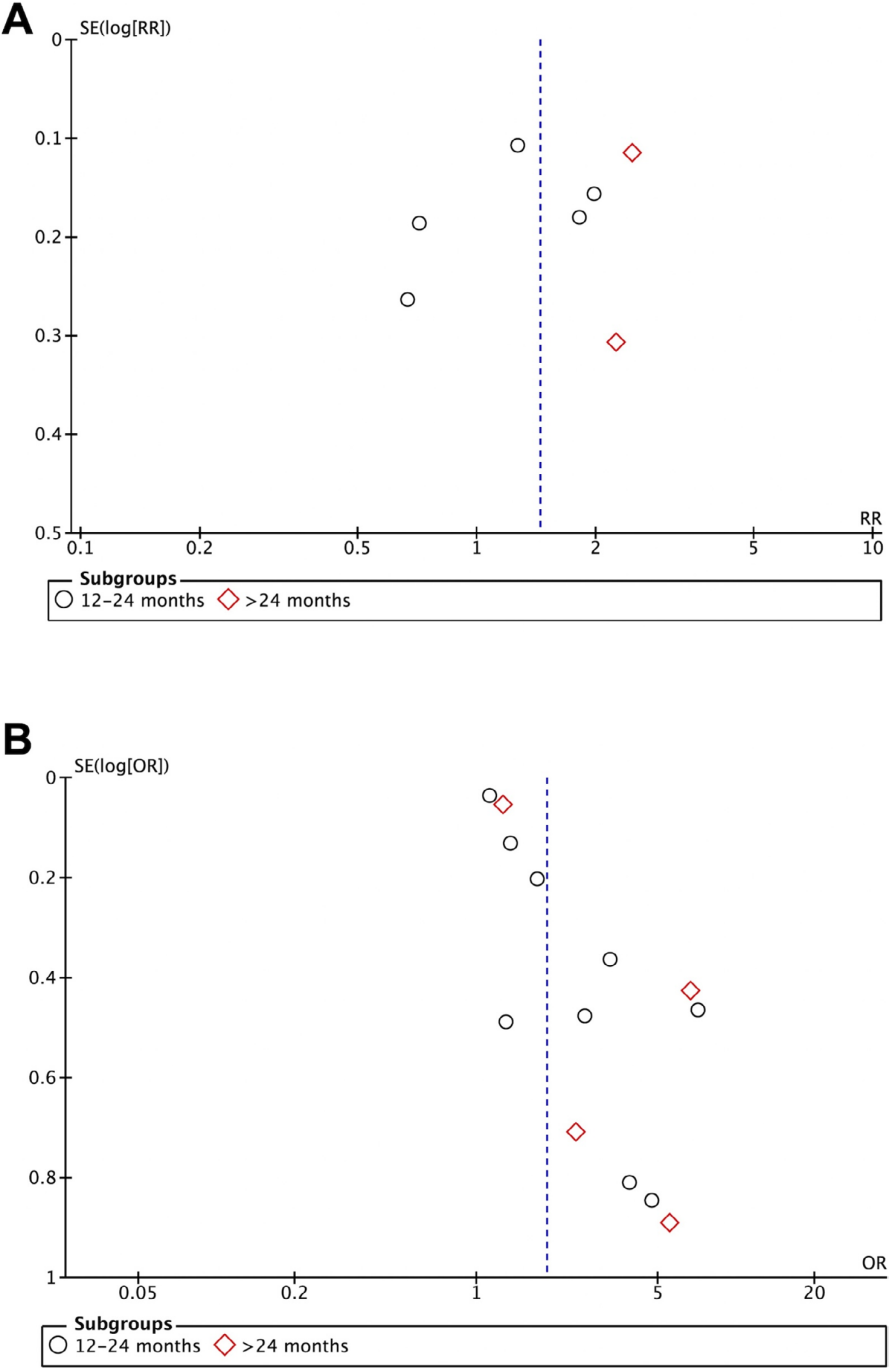
Funnel plot generated to assess potential publication bias. Figure (A) displays the studies presented in terms of relative risk, while figure (B) shows the prevalence studies.

Figure 6 shows the funnel plots of the studies included in the meta‐analysis. A visual inspection of the funnel plots suggested that publication bias was unlikely. Since fewer than 10 studies for each meta‐analysis were included, statistical tests for funnel plot asymmetry were not performed. Three non‐English studies were included in this analysis [26, 35, 43], 1 of which was sourced from the gray literature [43], thereby reducing the likelihood of publication bias.

### 3.6 Certainty of Evidence

The cumulative evidence was assessed using the GRADE criteria based on the primary outcome, “Risk of developing ECC associated with prolonged breastfeeding.” The overall evidence from cohort studies was judged to be low, with concerns in several domains: study design (very serious), as all studies were observational cohort studies; risk of bias (not serious), as most of the studies were classified as having a low risk of bias; and inconsistency (serious), due to significant heterogeneity among the studies, though the sensitivity analysis did not influence this heterogeneity. The following domains were not considered serious: indirectness, since the primary outcome of the selected studies matched the desirable outcome of this SR; and imprecision, due to substantial sample sizes with more than 300 participants per outcome. The evidence classification was upgraded due to a significant dose–response gradient found in the pooled results. A detailed summary of the findings using the GRADE approach is presented in Appendix S5.

## 4 Discussion

Currently, evidence on the risk of ECC associated with prolonged breastfeeding at 24 months or beyond remains limited [9, 12]. This review aimed to update the existing evidence on the relationship between breastfeeding and the risk of dental caries, with a particular focus on the impact of prolonged breastfeeding on ECC. The pooled data reviewed here suggest that breastfeeding beyond 24 months is a significant risk factor for ECC compared to non‐breastfed children, as shown by a previous systematic study [11]. Children breastfed for more than 24 months have nearly three times the odds of developing ECC compared to those not breastfed at the same age. Regarding breastfeeding between 12 and 24 months, this meta‐analysis found no significant risk of ECC, although the odds of ECC were notably higher during this period. This suggests no protective effect of breastfeeding against dental caries at this specific age.

The results of this review suggest that recommendations to maintain breastfeeding for 24 months or beyond, as advocated by the World Health Organization (WHO) and the United Nations Children's Fund (UNICEF), should be interpreted with caution. Despite the known benefits for children's general health and development [50, 51], this practice should be accompanied by parental guidance, dietary instructions regarding cariogenic foods, and efficient oral hygiene regimens due to the risk of dental caries. Dental caries not only affect dental health but also lead to other health consequences, such as pain, infection, altered eating and sleep habits, impaired cognitive development, reduced speech development, stunted growth, decreased concentration, and poor quality of life [52, 53]. Conversely, breastfeeding up to 12 months does not appear to pose a risk for dental caries and related comorbidities, as supported by previous reviews [9, 10, 11, 12], with a potential protective effect against dental caries [8, 10].

Shrestha et al. [12] found significant differences in dental caries between children breastfed for less than 12 months and those breastfed for 12 months or more, as well as between those breastfed for < 18 months and those breastfed for 18 months or more. They also reviewed three studies analyzing the influence of nocturnal breastfeeding on the risk of ECC compared to no nocturnal breastfeeding. Their findings suggest that breastfeeding for more than 12 months and nocturnal breastfeeding increase the risk of ECC.

The present meta‐analysis demonstrated an increased risk of ECC in children breastfed for more than 24 months and a higher odds of ECC between 12 and 24 months. However, nocturnal feeding habits were not assessed here due to the scarcity and heterogeneity of studies. This heterogeneity stems from the variety of feeding habits, including sugary drinks, bottle feeding, and breastfeeding, making it difficult to draw definitive conclusions. Only one study detailed nocturnal feeding habits in children beyond 18 months and was included in the qualitative analyses. According to their results, nocturnal feeding habits increase the risk of ECC related to prolonged breastfeeding [32].

A cariogenic diet is a well‐established risk factor for dental caries and may confound the association between prolonged breastfeeding and early childhood caries (ECC). Although most studies attempt to control for this and other confounding factors, variability in dietary habits and the methods used to collect dietary information—typically through caregiver interviews or questionnaires—remains a significant limitation inherent to observational studies. Some studies did not clearly describe how they accounted for the impact of cariogenic diets on ECC risk in samples of prolonged breastfeeding [34, 35, 36]. As a result, we were unable to fully evaluate the influence of diet on the risk of ECC in the context of prolonged breastfeeding. Similarly, oral hygiene practices were inconsistently analyzed across the included studies, introducing potential bias.

The Promotion of Breastfeeding Intervention Trial (PROBIT) [54] provides valuable insights as the only RCT conducted within the relevant timeframe to assess prolonged breastfeeding and reduce potential biases. This cluster‐randomized trial, which involved over 17 000 mother‐infant pairs, evaluated the effects of a breastfeeding promotion intervention on various health outcomes, including dental caries. While PROBIT significantly increased the prevalence and duration of exclusive breastfeeding, it found no significant association between prolonged or exclusive breastfeeding and dental caries at 6 years of age. These findings, based on an intention‐to‐treat analysis, suggest that prolonged breastfeeding neither has beneficial nor harmful effects on dental caries. Despite the important data that RCTs like PROBIT could provide for the present SR, the lack of additional RCTs limits the ability to conduct a pooled analysis that could offer generalizable results. Therefore, this review focused on observational cohort studies. The differences between the findings of this meta‐analysis and those of the PROBIT trial highlight the potential impact of methodological variations, confounding factors, and population differences.

In the context of this review, there were identified several limitations: a substantial number of studies did not report the method of dental caries diagnosis; there was a lack of data for calculating RR or OR; there were no reports on children's nocturnal feeding habits; some studies did not evaluate the influence of cariogenic diets alongside prolonged breastfeeding; and some studies did not assess the impact of oral hygiene frequency and habits on the risk of dental caries during prolonged breastfeeding. In addition, cumulative evidence from this study was based on findings from eight cohort studies with moderate overall quality of evidence.

Future studies should employ standardized and validated methods for diagnosing dental caries to ensure consistency and comparability across research. They should also include comprehensive dietary assessments that account for all types of food and drink consumption, particularly sugary and cariogenic foods, to control for dietary confounding factors. It is also important that these investigations evaluate the impact of different oral hygiene practices and regimens on the risk of ECC in breastfed children, considering factors such as brushing frequency, use of fluoride, and parental supervision. Additionally, they should investigate the specific impact of nocturnal breastfeeding and other nighttime feeding habits on ECC, with detailed data collection on the frequency and duration of nocturnal feedings.

This SR indicates that prolonged breastfeeding beyond 24 months significantly increases the risk of ECC, with a odds almost three times higher compared to non‐breastfed children, while breastfeeding between 12 and 24 months does not show a significant risk despite higher ECC odds. The findings suggest that recommendations for breastfeeding up to 24 months or beyond should be interpreted cautiously and supplemented with parental guidance, dietary instructions, and oral hygiene practices. The review highlights the need for future studies to use standardized diagnostic methods, comprehensive dietary assessments, and detailed evaluations of oral hygiene and nocturnal feeding habits to address current research limitations and better understand the relationship between prolonged breastfeeding and ECC.

## Author Contributions

In this study, the authors made the following contributions: K.L. and B.S.F.S. conceptualized the research design and methodology. L.R.S.R. and R.M.R. conducted data collection and analysis. T.P.P. contributed to the literature review and interpretation of results. E.M. provided critical insights and revisions for intellectual content. F.P.Y.S. contributed to experimental procedures and manuscript preparation. B.S.F.S. supervised the project and ensured the overall integrity and coherence of the study. All authors reviewed and approved the final manuscript.

## Conflicts of Interest

This study was partially financed by the Higher Education Personnel Improvement Coordenation ‐ Brazil (CAPES) [88887.675040/2022‐00].

## Supporting information


**Appendix** **S1**‐**S5**

## Data Availability

The data that supports the findings of this study are available in the Appendices S1–S5 of this article.
