# Supplementary material for: Risk of Early Childhood Dental Caries Associated With Prolonged Breastfeeding: A Systematic Review and Meta‐Analysis
Source: Int J Paediatr Dent. 2025 Apr 20;35(5):964–85. doi: 10.1111/ipd.13313 (PMC12332104; doi:10.1111/ipd.13313)
Supplement: Supplementary file 1 — Appendix S1‐S5 [file IPD-35-964-s001.docx]

**Appendix S1**. PRISMA 2020 Checklist.

| **Section and Topic** | **Item #** | **Checklist item** | **Location where item is reported** |
| --- | --- | --- | --- |
| **TITLE** | | |  |
| Title | 1 | Identify the report as a systematic review. | Page 1 |
| **ABSTRACT** | | |  |
| Abstract | 2 | See the PRISMA 2020 for Abstracts checklist. | Page 1 |
| **INTRODUCTION** | | |  |
| Rationale | 3 | Describe the rationale for the review in the context of existing knowledge. | Page 2 |
| Objectives | 4 | Provide an explicit statement of the objective(s) or question(s) the review addresses. | Page 3 |
| **METHODS** | | |  |
| Eligibility criteria | 5 | Specify the inclusion and exclusion criteria for the review and how studies were grouped for the syntheses. | Page 4 |
| Information sources | 6 | Specify all databases, registers, websites, organisations, reference lists and other sources searched or consulted to identify studies. Specify the date when each source was last searched or consulted. | Page 4 |
| Search strategy | 7 | Present the full search strategies for all databases, registers and websites, including any filters and limits used. | Appendix 1 |
| Selection process | 8 | Specify the methods used to decide whether a study met the inclusion criteria of the review, including how many reviewers screened each record and each report retrieved, whether they worked independently, and if applicable, details of automation tools used in the process. | Page 5 |
| Data collection process | 9 | Specify the methods used to collect data from reports, including how many reviewers collected data from each report, whether they worked independently, any processes for obtaining or confirming data from study investigators, and if applicable, details of automation tools used in the process. | Page 5 |
| Data items | 10a | List and define all outcomes for which data were sought. Specify whether all results that were compatible with each outcome domain in each study were sought (e.g. for all measures, time points, analyses), and if not, the methods used to decide which results to collect. | Page 6 |
|  | 10b | List and define all other variables for which data were sought (e.g. participant and intervention characteristics, funding sources). Describe any assumptions made about any missing or unclear information. | Page 6 |
| Study risk of bias assessment | 11 | Specify the methods used to assess risk of bias in the included studies, including details of the tool(s) used, how many reviewers assessed each study and whether they worked independently, and if applicable, details of automation tools used in the process. | Page 6 |
| Effect measures | 12 | Specify for each outcome the effect measure(s) (e.g. risk ratio, mean difference) used in the synthesis or presentation of results. | Page 6 |
| Synthesis methods | 13a | Describe the processes used to decide which studies were eligible for each synthesis (e.g. tabulating the study intervention characteristics and comparing against the planned groups for each synthesis (item #5)). | Page 6 |
|  | 13b | Describe any methods required to prepare the data for presentation or synthesis, such as handling of missing summary statistics, or data conversions. | Page 6 |
|  | 13c | Describe any methods used to tabulate or visually display results of individual studies and syntheses. | Page 7 |
|  | 13d | Describe any methods used to synthesize results and provide a rationale for the choice(s). If meta-analysis was performed, describe the model(s), method(s) to identify the presence and extent of statistical heterogeneity, and software package(s) used. | Page 7 |
|  | 13e | Describe any methods used to explore possible causes of heterogeneity among study results (e.g. subgroup analysis, meta-regression). | Page 7 |
|  | 13f | Describe any sensitivity analyses conducted to assess robustness of the synthesized results. | Page 6 |
| Reporting bias assessment | 14 | Describe any methods used to assess risk of bias due to missing results in a synthesis (arising from reporting biases). | Page 7 |
| Certainty assessment | 15 | Describe any methods used to assess certainty (or confidence) in the body of evidence for an outcome. | Page 7 |
| **RESULTS** | | |  |
| Study selection | 16a | Describe the results of the search and selection process, from the number of records identified in the search to the number of studies included in the review, ideally using a flow diagram. | Page 7 |
|  | 16b | Cite studies that might appear to meet the inclusion criteria, but which were excluded, and explain why they were excluded. | Page 7 |
| Study characteristics | 17 | Cite each included study and present its characteristics. | Page 8 |
| Risk of bias in studies | 18 | Present assessments of risk of bias for each included study. | Page 8 |
| Results of individual studies | 19 | For all outcomes, present, for each study: (a) summary statistics for each group (where appropriate) and (b) an effect estimate and its precision (e.g. confidence/credible interval), ideally using structured tables or plots. | Page 8 |
| Results of syntheses | 20a | For each synthesis, briefly summarise the characteristics and risk of bias among contributing studies. | Page 9 |
|  | 20b | Present results of all statistical syntheses conducted. If meta-analysis was done, present for each the summary estimate and its precision (e.g. confidence/credible interval) and measures of statistical heterogeneity. If comparing groups, describe the direction of the effect. | Page 9 |
|  | 20c | Present results of all investigations of possible causes of heterogeneity among study results. | Page 9 |
|  | 20d | Present results of all sensitivity analyses conducted to assess the robustness of the synthesized results. | Page 9 |
| Reporting biases | 21 | Present assessments of risk of bias due to missing results (arising from reporting biases) for each synthesis assessed. | Page 10 |
| Certainty of evidence | 22 | Present assessments of certainty (or confidence) in the body of evidence for each outcome assessed. | Page 10 |
| **DISCUSSION** | | |  |
| Discussion | 23a | Provide a general interpretation of the results in the context of other evidence. | Page 10 |
|  | 23b | Discuss any limitations of the evidence included in the review. | Page 12 |
|  | 23c | Discuss any limitations of the review processes used. | Page 12 |
|  | 23d | Discuss implications of the results for practice, policy, and future research. | Page 13 |
| **OTHER INFORMATION** | | |  |
| Registration and protocol | 24a | Provide registration information for the review, including register name and registration number, or state that the review was not registered. | Page 3 |
|  | 24b | Indicate where the review protocol can be accessed, or state that a protocol was not prepared. | Page 3 |
|  | 24c | Describe and explain any amendments to information provided at registration or in the protocol. | Page 3 |
| Support | 25 | Describe sources of financial or non-financial support for the review, and the role of the funders or sponsors in the review. | N/A |
| Competing interests | 26 | Declare any competing interests of review authors. | N/A |
| Availability of data, code and other materials | 27 | Report which of the following are publicly available and where they can be found: template data collection forms; data extracted from included studies; data used for all analyses; analytic code; any other materials used in the review. | N/A |

**Appendix S1**. PRISMA 2020 Checklist.

| **Section and Topic** | **Item #** | **Checklist item** | **Location where item is reported** |
| --- | --- | --- | --- |
| **TITLE** | | |  |
| Title | 1 | Identify the report as a systematic review. | Page 1 |
| **ABSTRACT** | | |  |
| Abstract | 2 | See the PRISMA 2020 for Abstracts checklist. | Page 1 |
| **INTRODUCTION** | | |  |
| Rationale | 3 | Describe the rationale for the review in the context of existing knowledge. | Page 2 |
| Objectives | 4 | Provide an explicit statement of the objective(s) or question(s) the review addresses. | Page 3 |
| **METHODS** | | |  |
| Eligibility criteria | 5 | Specify the inclusion and exclusion criteria for the review and how studies were grouped for the syntheses. | Page 4 |
| Information sources | 6 | Specify all databases, registers, websites, organisations, reference lists and other sources searched or consulted to identify studies. Specify the date when each source was last searched or consulted. | Page 4 |
| Search strategy | 7 | Present the full search strategies for all databases, registers and websites, including any filters and limits used. | Appendix 1 |
| Selection process | 8 | Specify the methods used to decide whether a study met the inclusion criteria of the review, including how many reviewers screened each record and each report retrieved, whether they worked independently, and if applicable, details of automation tools used in the process. | Page 5 |
| Data collection process | 9 | Specify the methods used to collect data from reports, including how many reviewers collected data from each report, whether they worked independently, any processes for obtaining or confirming data from study investigators, and if applicable, details of automation tools used in the process. | Page 5 |
| Data items | 10a | List and define all outcomes for which data were sought. Specify whether all results that were compatible with each outcome domain in each study were sought (e.g. for all measures, time points, analyses), and if not, the methods used to decide which results to collect. | Page 6 |
|  | 10b | List and define all other variables for which data were sought (e.g. participant and intervention characteristics, funding sources). Describe any assumptions made about any missing or unclear information. | Page 6 |
| Study risk of bias assessment | 11 | Specify the methods used to assess risk of bias in the included studies, including details of the tool(s) used, how many reviewers assessed each study and whether they worked independently, and if applicable, details of automation tools used in the process. | Page 6 |
| Effect measures | 12 | Specify for each outcome the effect measure(s) (e.g. risk ratio, mean difference) used in the synthesis or presentation of results. | Page 6 |
| Synthesis methods | 13a | Describe the processes used to decide which studies were eligible for each synthesis (e.g. tabulating the study intervention characteristics and comparing against the planned groups for each synthesis (item #5)). | Page 6 |
|  | 13b | Describe any methods required to prepare the data for presentation or synthesis, such as handling of missing summary statistics, or data conversions. | Page 6 |
|  | 13c | Describe any methods used to tabulate or visually display results of individual studies and syntheses. | Page 7 |
|  | 13d | Describe any methods used to synthesize results and provide a rationale for the choice(s). If meta-analysis was performed, describe the model(s), method(s) to identify the presence and extent of statistical heterogeneity, and software package(s) used. | Page 7 |
|  | 13e | Describe any methods used to explore possible causes of heterogeneity among study results (e.g. subgroup analysis, meta-regression). | Page 7 |
|  | 13f | Describe any sensitivity analyses conducted to assess robustness of the synthesized results. | Page 6 |
| Reporting bias assessment | 14 | Describe any methods used to assess risk of bias due to missing results in a synthesis (arising from reporting biases). | Page 7 |
| Certainty assessment | 15 | Describe any methods used to assess certainty (or confidence) in the body of evidence for an outcome. | Page 7 |
| **RESULTS** | | |  |
| Study selection | 16a | Describe the results of the search and selection process, from the number of records identified in the search to the number of studies included in the review, ideally using a flow diagram. | Page 7 |
|  | 16b | Cite studies that might appear to meet the inclusion criteria, but which were excluded, and explain why they were excluded. | Page 7 |
| Study characteristics | 17 | Cite each included study and present its characteristics. | Page 8 |
| Risk of bias in studies | 18 | Present assessments of risk of bias for each included study. | Page 8 |
| Results of individual studies | 19 | For all outcomes, present, for each study: (a) summary statistics for each group (where appropriate) and (b) an effect estimate and its precision (e.g. confidence/credible interval), ideally using structured tables or plots. | Page 8 |
| Results of syntheses | 20a | For each synthesis, briefly summarise the characteristics and risk of bias among contributing studies. | Page 9 |
|  | 20b | Present results of all statistical syntheses conducted. If meta-analysis was done, present for each the summary estimate and its precision (e.g. confidence/credible interval) and measures of statistical heterogeneity. If comparing groups, describe the direction of the effect. | Page 9 |
|  | 20c | Present results of all investigations of possible causes of heterogeneity among study results. | Page 9 |
|  | 20d | Present results of all sensitivity analyses conducted to assess the robustness of the synthesized results. | Page 9 |
| Reporting biases | 21 | Present assessments of risk of bias due to missing results (arising from reporting biases) for each synthesis assessed. | Page 10 |
| Certainty of evidence | 22 | Present assessments of certainty (or confidence) in the body of evidence for each outcome assessed. | Page 10 |
| **DISCUSSION** | | |  |
| Discussion | 23a | Provide a general interpretation of the results in the context of other evidence. | Page 10 |
|  | 23b | Discuss any limitations of the evidence included in the review. | Page 12 |
|  | 23c | Discuss any limitations of the review processes used. | Page 12 |
|  | 23d | Discuss implications of the results for practice, policy, and future research. | Page 13 |
| **OTHER INFORMATION** | | |  |
| Registration and protocol | 24a | Provide registration information for the review, including register name and registration number, or state that the review was not registered. | Page 3 |
|  | 24b | Indicate where the review protocol can be accessed, or state that a protocol was not prepared. | Page 3 |
|  | 24c | Describe and explain any amendments to information provided at registration or in the protocol. | Page 3 |
| Support | 25 | Describe sources of financial or non-financial support for the review, and the role of the funders or sponsors in the review. | N/A |
| Competing interests | 26 | Declare any competing interests of review authors. | N/A |
| Availability of data, code and other materials | 27 | Report which of the following are publicly available and where they can be found: template data collection forms; data extracted from included studies; data used for all analyses; analytic code; any other materials used in the review. | N/A |

**Appendix S2**. Databases and search strategies.

| Database | Search  strategy | Results  May 17^th^ 2024 |
| --- | --- | --- |
| Medline / PubMed | ("Breast Feeding"[MeSH Terms] OR "Breast Feeding"[All Fields] OR "Breastfed"[All Fields] OR "Breastfeeding"[All Fields] OR "Breast Fed"[All Fields] OR "Milk Sharing"[All Fields] OR "Exclusive Breast Feeding"[All Fields] OR "Exclusive Breastfeeding"[All Fields] OR "Wet Nursing"[All Fields] OR "sharing milk"[All Fields] OR "breast feeding exclusive"[All Fields] OR "breastfeeding exclusive"[All Fields] OR "Lactation"[MeSH Terms] OR "Lactation"[All Fields] OR "lactations"[All Fields] OR "Milk Secretion"[All Fields] OR "Milk Secretions"[All Fields] OR "Prolonged Lactation"[All Fields] OR "Prolonged Lactations"[All Fields] OR "Prolonged breast feeding"[All Fields] OR "Prolonged breastfeeding"[All Fields] OR "lactation prolonged"[All Fields] OR "milk, human"[MeSH Terms] OR "Breast Milk"[All Fields] OR "Human Milk"[All Fields] OR "milk breast"[All Fields] OR "Milk Secretion"[All Fields] OR "Milk Secretions"[All Fields] OR "Bottle Feeding"[MeSH Terms] OR "Bottle Feeding"[All Fields] OR "Bottlefeeding"[All Fields] OR "Bottlefed"[All Fields]) AND ("Dental Caries"[MeSH Terms] OR "Dental Caries"[All Fields] OR "Dental Cavity"[All Fields] OR "Dental Decay"[All Fields] OR "Dental Cavities"[All Fields] OR "Carious Lesions"[All Fields] OR "Carious Lesion"[All Fields] OR "Carious Dentin"[All Fields] OR "Dental White Spot"[All Fields] OR "Dental White Spots"[All Fields] OR "caries dental"[All Fields] OR "cavities dental"[All Fields] OR "cavity dental"[All Fields] OR "lesions carious"[All Fields] OR "decay dental"[All Fields] OR "dentin carious"[All Fields] OR "white spot dental"[All Fields] OR "Early childhood caries"[All Fields] OR "Tooth Demineralization"[MeSH Terms] OR "Tooth Demineralization"[All Fields]) | 978 |
| Embase | ('breast feeding'/de OR 'breast feeding' OR breastfed OR 'breastfeeding'/de OR breastfeeding OR 'breast fed' OR 'milk sharing' OR 'exclusive breast feeding'/de OR 'exclusive breast feeding' OR 'exclusive breastfeeding'/de OR 'exclusive breastfeeding' OR 'wet nursing' OR 'sharing milk' OR 'breast feeding exclusive' OR 'breastfeeding exclusive' OR 'lactation'/de OR lactation OR lactations OR 'prolonged lactation'/de OR 'prolonged lactation' OR 'prolonged lactations' OR 'prolonged breast feeding' OR 'prolonged breastfeeding' OR 'lactation prolonged'/de OR 'lactation prolonged' OR 'breast milk'/de OR 'breast milk' OR 'human milk'/de OR 'human milk' OR 'milk breast' OR 'milk secretion'/de OR 'milk secretion' OR 'milk secretions' OR 'bottle feeding'/de OR 'bottle feeding' OR bottlefeeding OR bottlefed) AND ('dental caries'/de OR 'dental caries' OR 'dental cavity' OR 'dental decay'/de OR 'dental decay' OR 'dental cavities'/de OR 'dental cavities' OR 'carious lesions' OR 'carious lesion' OR 'carious dentin' OR 'dental white spot' OR 'dental white spots' OR 'caries dental'/de OR 'caries dental' OR 'cavities dental' OR 'cavity dental' OR 'lesions carious' OR 'decay dental' OR 'dentin carious' OR 'white spot dental' OR 'early childhood caries'/de OR 'early childhood caries' OR 'tooth demineralization'/de OR 'tooth demineralization') | 1,128 |
| Scopus | TITLE-ABS-KEY("Breast Feeding" OR Breastfed OR Breastfeeding OR "Breast Fed" OR "Milk Sharing" OR "Exclusive Breast Feeding" OR "Exclusive Breastfeeding" OR "Wet Nursing" OR "sharing milk" OR "breast feeding exclusive" OR "breastfeeding exclusive" OR Lactation OR lactations OR "Milk Secretion" OR "Milk Secretions" OR "Prolonged Lactation" OR "Prolonged Lactations" OR "Prolonged breast feeding" OR "Prolonged breastfeeding" OR "lactation prolonged" OR "Breast Milk" OR "Human Milk" OR "milk breast" OR "Milk Secretion" OR "Milk Secretions" OR "Bottle Feeding" OR Bottlefeeding OR Bottlefed) AND TITLE-ABS-KEY("Dental Caries" OR "Dental Cavity" OR "Dental Decay" OR "Dental Cavities" OR "Carious Lesions" OR "Carious Lesion" OR "Carious Dentin" OR "Dental White Spot" OR "Dental White Spots" OR "caries dental" OR "cavities dental" OR "cavity dental" OR "lesions carious" OR "decay dental" OR "dentin carious" OR "white spot dental" OR "Early childhood caries" OR "Tooth Demineralization") | 1,191 |
| Web of Science | TS=("Breast Feeding" OR Breastfed OR Breastfeeding OR "Breast Fed" OR "Milk Sharing" OR "Exclusive Breast Feeding" OR "Exclusive Breastfeeding" OR "Wet Nursing" OR "sharing milk" OR "breast feeding exclusive" OR "breastfeeding exclusive" OR Lactation OR lactations OR "Milk Secretion" OR "Milk Secretions" OR "Prolonged Lactation" OR "Prolonged Lactations" OR "Prolonged breast feeding" OR "Prolonged breastfeeding" OR "lactation prolonged" OR "Breast Milk" OR "Human Milk" OR "milk breast" OR "Milk Secretion" OR "Milk Secretions" OR "Bottle Feeding" OR Bottlefeeding OR Bottlefed) AND TS=("Dental Caries" OR "Dental Cavity" OR "Dental Decay" OR "Dental Cavities" OR "Carious Lesions" OR "Carious Lesion" OR "Carious Dentin" OR "Dental White Spot" OR "Dental White Spots" OR "caries dental" OR "cavities dental" OR "cavity dental" OR "lesions carious" OR "decay dental" OR "dentin carious" OR "white spot dental" OR "Early childhood caries" OR "Tooth Demineralization") | 390 |
| Cochrane Library | ("Breast Feeding" OR Breastfed OR Breastfeeding OR "Breast Fed" OR "Milk Sharing" OR "Exclusive Breast Feeding" OR "Exclusive Breastfeeding" OR "Wet Nursing" OR "sharing milk" OR "breast feeding exclusive" OR "breastfeeding exclusive" OR Lactation OR lactations OR "Milk Secretion" OR "Milk Secretions" OR "Prolonged Lactation" OR "Prolonged Lactations" OR "Prolonged breast feeding" OR "Prolonged breastfeeding" OR "lactation prolonged" OR "Breast Milk" OR "Human Milk" OR "milk breast" OR "Milk Secretion" OR "Milk Secretions" OR "Bottle Feeding" OR Bottlefeeding OR Bottlefed):ti,ab,kw AND ("Dental Caries" OR "Dental Cavity" OR "Dental Decay" OR "Dental Cavities" OR "Carious Lesions" OR "Carious Lesion" OR "Carious Dentin" OR "Dental White Spot" OR "Dental White Spots" OR "caries dental" OR "cavities dental" OR "cavity dental" OR "lesions carious" OR "decay dental" OR "dentin carious" OR "white spot dental" OR "Early childhood caries" OR "Tooth Demineralization"):ti,ab,kw | 56 |
| CINAHL (EBSCO) | (TI("Breast Feeding" OR Breastfed OR Breastfeeding OR "Breast Fed" OR "Milk Sharing" OR "Exclusive Breast Feeding" OR "Exclusive Breastfeeding" OR "Wet Nursing" OR "sharing milk" OR "breast feeding exclusive" OR "breastfeeding exclusive" OR Lactation OR lactations OR "Milk Secretion" OR "Milk Secretions" OR "Prolonged Lactation" OR "Prolonged Lactations" OR "Prolonged breast feeding" OR "Prolonged breastfeeding" OR "lactation prolonged" OR "Breast Milk" OR "Human Milk" OR "milk breast" OR "Milk Secretion" OR "Milk Secretions" OR "Bottle Feeding" OR Bottlefeeding OR Bottlefed) AND TI("Dental Caries" OR "Dental Cavity" OR "Dental Decay" OR "Dental Cavities" OR "Carious Lesions" OR "Carious Lesion" OR "Carious Dentin" OR "Dental White Spot" OR "Dental White Spots" OR "caries dental" OR "cavities dental" OR "cavity dental" OR "lesions carious" OR "decay dental" OR "dentin carious" OR "white spot dental" OR "Early childhood caries" OR "Tooth Demineralization")) OR (AB("Breast Feeding" OR Breastfed OR Breastfeeding OR "Breast Fed" OR "Milk Sharing" OR "Exclusive Breast Feeding" OR "Exclusive Breastfeeding" OR "Wet Nursing" OR "sharing milk" OR "breast feeding exclusive" OR "breastfeeding exclusive" OR Lactation OR lactations OR "Milk Secretion" OR "Milk Secretions" OR "Prolonged Lactation" OR "Prolonged Lactations" OR "Prolonged breast feeding" OR "Prolonged breastfeeding" OR "lactation prolonged" OR "Breast Milk" OR "Human Milk" OR "milk breast" OR "Milk Secretion" OR "Milk Secretions" OR "Bottle Feeding" OR Bottlefeeding OR Bottlefed) AND AB("Dental Caries" OR "Dental Cavity" OR "Dental Decay" OR "Dental Cavities" OR "Carious Lesions" OR "Carious Lesion" OR "Carious Dentin" OR "Dental White Spot" OR "Dental White Spots" OR "caries dental" OR "cavities dental" OR "cavity dental" OR "lesions carious" OR "decay dental" OR "dentin carious" OR "white spot dental" OR "Early childhood caries" OR "Tooth Demineralization")) OR (SU("Breast Feeding" OR Breastfed OR Breastfeeding OR "Breast Fed" OR "Milk Sharing" OR "Exclusive Breast Feeding" OR "Exclusive Breastfeeding" OR "Wet Nursing" OR "sharing milk" OR "breast feeding exclusive" OR "breastfeeding exclusive" OR Lactation OR lactations OR "Milk Secretion" OR "Milk Secretions" OR "Prolonged Lactation" OR "Prolonged Lactations" OR "Prolonged breast feeding" OR "Prolonged breastfeeding" OR "lactation prolonged" OR "Breast Milk" OR "Human Milk" OR "milk breast" OR "Milk Secretion" OR "Milk Secretions" OR "Bottle Feeding" OR Bottlefeeding OR Bottlefed) AND SU("Dental Caries" OR "Dental Cavity" OR "Dental Decay" OR "Dental Cavities" OR "Carious Lesions" OR "Carious Lesion" OR "Carious Dentin" OR "Dental White Spot" OR "Dental White Spots" OR "caries dental" OR "cavities dental" OR "cavity dental" OR "lesions carious" OR "decay dental" OR "dentin carious" OR "white spot dental" OR "Early childhood caries" OR "Tooth Demineralization")) | 300 |
| LILACS /  via BVS | ("Breast Feeding" OR breastfed OR breastfeeding OR "Breast Fed" OR "Milk Sharing" OR "Exclusive Breast Feeding" OR "Exclusive Breastfeeding" OR "Wet Nursing" OR "sharing milk" OR "breast feeding exclusive" OR "breastfeeding exclusive" OR lactation OR lactations OR "Milk Secretion" OR "Milk Secretions" OR "Prolonged Lactation" OR "Prolonged Lactations" OR "Prolonged breast feeding" OR "Prolonged breastfeeding" OR "lactation prolonged" OR "Breast Milk" OR "Human Milk" OR "milk breast" OR "Milk Secretion" OR "Milk Secretions" OR "Bottle Feeding" OR bottlefeeding OR bottlefed OR "Aleitamento Materno" OR aleitamento OR "Aleitamento Materno Exclusivo" OR "Alimentado ao Peito" OR "Alimentado no Peito" OR "Alimentação ao Peito" OR amamentado OR amamentação OR "Amamentação com Ama-de-Leite" OR "Compartilhamento de Leite" OR "Lactancia Materna" OR "Alimentación al Pecho" OR amamantado OR amamantamiento OR "Compartir Leche" OR "Enfermería Húmeda" OR "alimentación por nodriza" OR "compartir leche materna" OR lactação OR "Lactação Prolongada" OR "Secreção de Leite" OR "Secreções de Leite" OR lactancia OR "Lactancia Prolongada" OR "Secreciones de Leche" OR "Secreción de Leche" OR "Leite Humano" OR "Leite Materno" OR "Leche Humana" OR "Leche Materna" OR "Leche de la Madre" OR "Alimentação com Mamadeira" OR "Aleitamento por Mamadeira" OR "Alimentado por Mamadeira" OR "Alimentación con Biberón" OR "Alimentado con Biberón" OR "alimentación por biberón" ) AND ("Dental Caries" OR "Dental Cavity" OR "Dental Decay" OR "Dental Cavities" OR "Carious Lesions" OR "Carious Lesion" OR "Carious Dentin" OR "Dental White Spot" OR "Dental White Spots" OR "caries dental" OR "cavities dental" OR "cavity dental" OR "lesions carious" OR "decay dental" OR "dentin carious" OR "white spot dental" OR "Early childhood caries" OR "Tooth Demineralization" OR "Cárie Dentária" OR "Cavidade Dentária" OR "Cavidades Dentárias" OR "Cárie Dental" OR caries OR "Cáries Dentais" OR "Cáries Dentárias" OR "Dente Cariado" OR "Lesões Cariosas" OR "Manchas (hipocalcificadas) Brancas Dentárias" OR "Manchas Brancas" OR "Caries Dental" OR "Caries Dentales" OR "Cavidad Dental" OR "Cavidades Dentales" OR "Lesiones Cariosas" OR "Manchas Blancas Dentales" OR "caries dentaria" OR "manchas blancas del esmalte" OR "Carious Dentins" OR "Desmineralização do Dente" OR "Desmineralização Dentária" OR "Desmineralizações dos Dentes" OR "Desmineralización Dental") AND ( db:("LILACS")) | 210 |
| Bibliografia Brasileira de Odontologia (BBO) / via BVS | ("Breast Feeding" OR breastfed OR breastfeeding OR "Breast Fed" OR "Milk Sharing" OR "Exclusive Breast Feeding" OR "Exclusive Breastfeeding" OR "Wet Nursing" OR "sharing milk" OR "breast feeding exclusive" OR "breastfeeding exclusive" OR lactation OR lactations OR "Milk Secretion" OR "Milk Secretions" OR "Prolonged Lactation" OR "Prolonged Lactations" OR "Prolonged breast feeding" OR "Prolonged breastfeeding" OR "lactation prolonged" OR "Breast Milk" OR "Human Milk" OR "milk breast" OR "Milk Secretion" OR "Milk Secretions" OR "Bottle Feeding" OR bottlefeeding OR bottlefed OR "Aleitamento Materno" OR aleitamento OR "Aleitamento Materno Exclusivo" OR "Alimentado ao Peito" OR "Alimentado no Peito" OR "Alimentação ao Peito" OR amamentado OR amamentação OR "Amamentação com Ama-de-Leite" OR "Compartilhamento de Leite" OR "Lactancia Materna" OR "Alimentación al Pecho" OR amamantado OR amamantamiento OR "Compartir Leche" OR "Enfermería Húmeda" OR "alimentación por nodriza" OR "compartir leche materna" OR lactação OR "Lactação Prolongada" OR "Secreção de Leite" OR "Secreções de Leite" OR lactancia OR "Lactancia Prolongada" OR "Secreciones de Leche" OR "Secreción de Leche" OR "Leite Humano" OR "Leite Materno" OR "Leche Humana" OR "Leche Materna" OR "Leche de la Madre" OR "Alimentação com Mamadeira" OR "Aleitamento por Mamadeira" OR "Alimentado por Mamadeira" OR "Alimentación con Biberón" OR "Alimentado con Biberón" OR "alimentación por biberón" ) AND ("Dental Caries" OR "Dental Cavity" OR "Dental Decay" OR "Dental Cavities" OR "Carious Lesions" OR "Carious Lesion" OR "Carious Dentin" OR "Dental White Spot" OR "Dental White Spots" OR "caries dental" OR "cavities dental" OR "cavity dental" OR "lesions carious" OR "decay dental" OR "dentin carious" OR "white spot dental" OR "Early childhood caries" OR "Tooth Demineralization" OR "Cárie Dentária" OR "Cavidade Dentária" OR "Cavidades Dentárias" OR "Cárie Dental" OR caries OR "Cáries Dentais" OR "Cáries Dentárias" OR "Dente Cariado" OR "Lesões Cariosas" OR "Manchas (hipocalcificadas) Brancas Dentárias" OR "Manchas Brancas" OR "Caries Dental" OR "Caries Dentales" OR "Cavidad Dental" OR "Cavidades Dentales" OR "Lesiones Cariosas" OR "Manchas Blancas Dentales" OR "caries dentaria" OR "manchas blancas del esmalte" OR "Carious Dentins" OR "Desmineralização do Dente" OR "Desmineralização Dentária" OR "Desmineralizações dos Dentes" OR "Desmineralización Dental") AND ( db:("BBO")) | 120 |
| ProQuest Dissertations & Theses Global | noft("Breast Feeding" OR Breastfed OR Breastfeeding OR "Breast Fed" OR "Milk Sharing" OR "Exclusive Breast Feeding" OR "Exclusive Breastfeeding" OR "Wet Nursing" OR "sharing milk" OR "breast feeding exclusive" OR "breastfeeding exclusive" OR Lactation OR lactations OR "Milk Secretion" OR "Milk Secretions" OR "Prolonged Lactation" OR "Prolonged Lactations" OR "Prolonged breast feeding" OR "Prolonged breastfeeding" OR "lactation prolonged" OR "Breast Milk" OR "Human Milk" OR "milk breast" OR "Milk Secretion" OR "Milk Secretions" OR "Bottle Feeding" OR Bottlefeeding OR Bottlefed) AND noft("Dental Caries" OR "Dental Cavity" OR "Dental Decay" OR "Dental Cavities" OR "Carious Lesions" OR "Carious Lesion" OR "Carious Dentin" OR "Dental White Spot" OR "Dental White Spots" OR "caries dental" OR "cavities dental" OR "cavity dental" OR "lesions carious" OR "decay dental" OR "dentin carious" OR "white spot dental" OR "Early childhood caries" OR "Tooth Demineralization") | 36 |
| LIVIVO | ("Breast Feeding" OR Breastfed OR Breastfeeding OR "Breast Fed" OR "Milk Sharing" OR "Exclusive Breast Feeding" OR "Exclusive Breastfeeding" OR "Wet Nursing" OR "sharing milk" OR "breast feeding exclusive" OR "breastfeeding exclusive" OR Lactation OR lactations OR "Milk Secretion" OR "Milk Secretions" OR "Prolonged Lactation" OR "Prolonged Lactations" OR "Prolonged breast feeding" OR "Prolonged breastfeeding" OR "lactation prolonged" OR "Breast Milk" OR "Human Milk" OR "milk breast" OR "Milk Secretion" OR "Milk Secretions" OR "Bottle Feeding" OR Bottlefeeding OR Bottlefed) AND ("Dental Caries" OR "Dental Cavity" OR "Dental Decay" OR "Dental Cavities" OR "Carious Lesions" OR "Carious Lesion" OR "Carious Dentin" OR "Dental White Spot" OR "Dental White Spots" OR "caries dental" OR "cavities dental" OR "cavity dental" OR "lesions carious" OR "decay dental" OR "dentin carious" OR "white spot dental" OR "Early childhood caries" OR "Tooth Demineralization") | 480 |
| Google Scholar | ("Breast Feeding" OR Breastfeeding OR Lactation OR "Breast Milk" OR "Bottle Feeding" OR Bottlefeeding) AND ("Dental Caries" OR "Dental Cavity" OR "Dental Decay" OR "Dental Cavities") | 100 |

Search strategies were performed for each database by using specifics words combinations and truncations with the support of a librarian.

**Appendix S3.** Excluded articles and reasons for exclusion (n=20).

| **Author, Year** | **Reason for exclusion** |
| --- | --- |
| Beckett *et al.,* 2022 | 2 |
| Bell et al., 2019 | 4 |
| Boustedt et al., 2018 | 3 |
| Cangussu et al., 2016 | 3 |
| Chattopadhyay et al., 2020 | 3 |
| Conway, 2023 | 6 |
| de Sousa Cabral et al., 2017 | 3 |
| Kuhn & Stadler, 2007 | 3 |
| Ha et al., 2023 | 3 |
| Hu et al., 2019 | 3 |
| Peltzer et al., 2014 | 4 |
| Lam et al., 2017 | 4 |
| Mizoguchi et al., 2003 | 3 |
| Nakai & Mori-Suzuki, 2022 | 6 |
| Nishimura et al., 2008 | 6 |
| Olatosi et al., 2021 | 3 |
| Schwarz et al., 2011 | 4 |
| Tiberia et al., 2007 | 3 |
| Uerlich et al., 2021 | 3 |
| Yonezu & Yakushiji, 2008 | 4 |
| (1) children with known immunological diseases, with physical, neurological or metabolic syndromes, with chronic history of infection, or with history of preterm birth (< 36 weeks); (2) children over 71 months old; (3) other types of feeding practices; (4) absence of the comparator group or N/A; (5) dental hypoplasia and other dental abnormalities; (6) *in vitro* studies, animal studies, case reports, cross-sectional, case-control, randomized trial, reviews, letters, personal opinions, book chapters, and conference abstracts. | |

**Appendix S4.** Results from Joanna Briggs Institute Critical Appraisal Checklist for cohort studies (*Moola S, Munn Z, Tufanaru C, Aromataris E, Sears K, Sfetcu R, Currie M, Qureshi R, Mattis P, Lisy K, Mu P-F. Chapter 7: Systematic reviews of etiology and risk . In: Aromataris E, Munn Z (Editors). JBI Manual for Evidence Synthesis. JBI, 2020. Available from https://synthesismanual.jbi.global*).

| **Author, year** | **Q1** | **Q2** | **Q3** | **Q4** | **Q5** | **Q6** | **Q7** | **Q8** | **Q9** | **Q10** | **Q11** | **Total**  **ƩY** | **Risk of Bias** |
| --- | --- | --- | --- | --- | --- | --- | --- | --- | --- | --- | --- | --- | --- |
| Abanto et al., 2022. | Y | Y | U | Y | Y | Y | Y | Y | Y | NA | Y | 90% | Low |
| Barroso et al., 2021 | Y | Y | U | Y | Y | Y | U | Y | N | N | Y | 63% | Mod |
| Bernabé et al., 2016 | NA | NA | U | Y | N | Y | U | Y | Y | NA | Y | 72% | Low |
| Birungi et al., 2017 | U | U | U | Y | Y | Y | Y | Y | Y | NA | Y | 72% | Low |
| Blanco et al., 2021 | U | U | U | Y | N | Y | U | Y | Y | NA | Y | 54% | Mod |
| Chaffee et al., 2014 | U | U | U | Y | N | Y | U | Y | Y | NA | Y | 54% | Mod |
| Devenish et al., 2020 | Y | Y | Y | Y | Y | Y | U | Y | N | N | Y | 72% | Low |
| Felden et al.,2010. | Y | Y | Y | Y | Y | Y | Y | Y | Y | NA | Y | 100% | Low |
| Feldens et al., 2018 | Y | Y | Y | Y | Y | Y | Y | Y | Y | N | Y | 90% | Low |
| Helderman et al., 2006. | Y | Y | Y | Y | Y | Y | Y | Y | Y | NA | Y | 100% | Low |
| Hong et al., 2014. | Y | Y | U | N | NA | Y | U | Y | Y | NA | Y | 72% | Low |
| Ibrahim et al., 2009. | Y | Y | Y | N | NA | Y | U | Y | Y | NA | Y | 81% | Low |
| Ji et al., 2006 | Y | Y | N | Y | N | Y | U | Y | Y | U | Y | 63% | Mod |
| Lunteren et al., 2020 | Y | Y | Y | Y | Y | U | Y | Y | Y | NA | Y | 90% | Low |
| Mathias et al., 2023 | N | Y | Y | Y | Y | Y | Y | Y | Y | NA | Y | 90% | Low |
| Nakamura, 2009. | Y | Y | Y | U | U | Y | Y | Y | Y | NA | Y | 81% | Low |
| Nirunsittirat et al., 2016 | Y | Y | Y | Y | Y | Y | Y | Y | Y | NA | Y | 100% | Low |
| Nunes et al., 2012 | Y | Y | Y | Y | Y | Y | Y | Y | Y | NA | Y | 100% | Low |
| Peres et al., 2017 | Y | Y | Y | Y | Y | Y | Y | Y | Y | NA | Y | 100% | Low |
| Pires et al., 2020 | Y | Y | Y | Y | Y | Y | Y | Y | Y | NA | Y | 100% | Low |
| Sæthre, Wang & Wigen, 2023. | Y | Y | Y | Y | Y | Y | Y | Y | Y | NA | Y | 100% | Low |
| Sritangsirikul et al., 2024 | Y | Y | Y | Y | Y | Y | Y | Y | Y | Y | Y | 100% | Low |
| Tada et al., 1999 | Y | U | Y | Y | Y | Y | Y | Y | Y | NA | Y | 90% | Low |
| Tashiro et al. 2021 | Y | Y | Y | Y | Y | Y | Y | Y | Y | NA | Y | 100% | Low |
| Yokoi et al. 2021 | Y | Y | Y | Y | Y | Y | Y | Y | Y | NA | Y | 100% | Low |

*Y* yes, *N* no, *U* unclear, *NA* not applicable

Q1. Were the two groups similar and recruited from the same population?

Q2. Were the exposures measured similarly to assign people to both exposed and unexposed groups?

Q3. Was the exposure measured in a valid and reliable way?

Q4. Were confounding factors identified?

Q5. Were strategies to deal with confounding factors stated?

Q6. Were the groups/participants free of the outcome at the start of the study (or at the moment of exposure)?

Q7. Were the outcomes measured in a valid and reliable way?

Q8. Was the follow up time reported and sufficient to be long enough for outcomes to occur?

Q9. Was follow up complete, and if not, were the reasons to loss to follow up described and explored?

Q10. Were strategies to address incomplete follow up utilized?

Q11. Was appropriate statistical analysis used?

Total = ΣY/applicable items (the not applicable (NA) items were excluded from the sum)

Risk of bias (methodologic quality) was categorized as high when the study reaches up to 49% score “yes”, moderate when the study reached 50 to 69% score “yes”, and low when the study reached more than 70% score “yes”

**Appendix S5.** GRADE

**Question**: What is the risk of ECC associated to prolonged breastfeeding?

| **Certainty assessment** | | | | | | | | | | | **Sample** | | **Effect** | | **Certainty** | **Importance** |
| --- | --- | --- | --- | --- | --- | --- | --- | --- | --- | --- | --- | --- | --- | --- | --- | --- |
| **№ of studies** | **Study design** | | **Risk of bias** | **Inconsistency** | | **Indirectness** | | **Imprecision** | **Other considerations** | | **With ECC Exposed to prolonged breastfeeding** | **With ECC Not Exposed to prolonged breastfeeding** | **Relative**  **(IC 95%)** | **Absolut**  **(IC 95%)** |  |  |
| **Risk of developing caries associated with prolonged breastfeeding** | | | | | | | | | | | | | | | | |
| 7 | observational studies - cohort | | not serious | serious | | not serious | | not serious | dose response gradient | | 900/2194 (41.0%) | 1872/6257  (29.9%) | RR 1.44  (1.01 - 2.07) | 132 per  1.000 | ⨁⨁⨁◯  Moderate | CRITICAL |
|  | |  | | |  | |  | | |  | | | | | | |

**References**

Beckett DM, Wheeler BJ, Loch C, Mahoney EK, Drummond BK, Broadbent JM. Dental health in a cohort of six-year-old New Zealand children who were breastfed as infants – a comprehensive descriptive study. N Z Dent J. 2022;118(1):5-13.

Bell LK, Schammer C, Devenish G, Ha D, Thomson MW, Spencer JA, et al. Dietary patterns and risk of obesity and early childhood caries in Australian toddlers: Findings from an Australian cohort study. Nutrients. 2019;11(11):2828

Boustedt K, Roswall J, Twetman S, Dahlgren J. Influence of mode of delivery, family and nursing determinants on early childhood caries development: a prospective cohort study. Acta Odontol Scand. 2018;76(8):595-9.

Cangussu MC, Cabral MB, Mota EL, Vianna MI. Fatores de risco para a cárie dental em crianças na primeira infância, Salvador – BA. Rev Bras Saude Matern Infant. 2016;16(1):57-65.

Chattopadhyay A, Christian B, Masood M, Calache H, Carpenter L, Gibbs L, et al. Natural history of dental caries: Baseline characteristics of the VicGen birth cohort study. Int J Paediatr Dent. 2020;30(3):334-41.

Conway F. Is prolonged breastfeeding a risk factor for dental caries at 2 years of age? Evid Based Dent. 2023;24(1):30-1.

de Sousa Cabral MB, Mota ELA, Cangussu MC, Vianna MI, Floriano FR. Risk factors for caries-free time: longitudinal study in early childhood. Rev Saude Publica. 2017;51:1-12.

Kuhn E, Stadler DS. Incidence of Caries in Babies After 15 Months of an Educative/Preventive Program. Pesqui Bras Odontopediatria Clin Integr. 2007;7(1):75-81.

Ha EK, Kim JH, Cha HR, Lee WS, Lee SW, Han MY. Relationship Between Feeding to Sleep During Infancy and Subsequent Childhood Disease Burden. J Pediatr. 2023;256:85-91.e3.

Hu S, Sim YF, Toh JY, Saw SM, Godfrey KM, Chong YS, et al. Infant dietary patterns and early childhood caries in a multi-ethnic Asian cohort. Sci Rep. 2019;9(1):1-8.

Peltzer K, Mongkolchati A, Satchaiyan G, Rajchagool T, Pimpak T. Sociobehavioral Factors Associated with Caries Increment: A Longitudinal Study from 24 to 36 Months Old Children in Thailand. Int J Environ Res Public Health. 2014;11(10):10838-50.

Lam CU, Khin LW, Kalhan AC, Yee R, Lee YS, Chong MF, et al. Identification of Caries Risk Determinants in Toddlers: Results of the GUSTO Birth Cohort Study. Caries Res. 2017;51(4):271-82.

Mizoguchi K, Kurumado K, Tango T, Minowa M. Study on factors for caries and infant feeding characteristics in children aged 1.5-3 years in a Kanto urban area. Nihon Koshu Eisei Zasshi. 2003;50(9):867-78.

Nakai Y, Mori-Suzuki Y. Impact of Dietary Patterns on Plaque Acidogenicity and Dental Caries in Early Childhood: A Retrospective Analysis in Japan. Int J Environ Res Public Health. 2022;19(12): 7245.

Nishimura M, Oda T, Kariya N, Matsumura S, Shimono T. Using a caries activity test to predict caries risk in early childhood. J Am Dent Assoc. 2008;139(1):63-71.

Olatosi OO, Li M, Alade AA, Oyapero A, Busch T, Pape J, et al. Replication of GWAS significant loci in a sub-Saharan African cohort with early childhood caries: a pilot study. BMC Oral Health. 2021;21(1):274.

Schwarz E, Do L, Bhole S, Scott JA, Arora A, Blinkhorn AS. Early childhood feeding practices and dental caries in preschool children: a multi-centre birth cohort study. BMC Public Health. 2011;11(1):28.

Tiberia MJ, Milnes AR, Feigal RJ, Morley KR, Richardson DS, Croft WG, et al. Risk factors for early childhood caries in Canadian preschool children seeking care. Pediatr Dent. 2007;29(3):201-8.

Uerlich MF, Baker SR, Day PF, Brown L, Vettore MV. Common determinants of dental caries and obesity in children: A multi-ethnic nested birth cohort study in the United Kingdom. Int J Environ Res Public Health. 2021;18(23):12561.

Yonezu T, Yakushiji M. Longitudinal study on influence of prolonged non-nutritive sucking habits on dental caries in Japanese children from 1.5 to 3 years of age. Bull Tokyo Dent Coll. 2008;49(2):59-63.
